# *In Vitro* Mutational Analysis of the β_2_ Adrenergic Receptor, an *In Vivo* Surrogate Odorant Receptor

**DOI:** 10.1371/journal.pone.0141696

**Published:** 2015-10-29

**Authors:** Sophie Jamet, Jaclyn Bubnell, Patrick Pfister, Delia Tomoiaga, Matthew E. Rogers, Paul Feinstein

**Affiliations:** 1 Department of Biological Sciences, Hunter College, CUNY, New York, New York, United States of America; 2 The Graduate Center Biology Program, CUNY, New York, New York, United States of America; 3 The Graduate Center Behavioral and Cognitive Neuroscience Program, CUNY, New York, New York, United States of America; 4 Corporate Research and Development, Firmenich Inc., Plainsboro, New Jersey, United States of America; Monell Chemical Senses Center, UNITED STATES

## Abstract

Many G-protein coupled receptors (GPCRs), such as odorant receptors (ORs), cannot be characterized in heterologous cells because of their difficulty in trafficking to the plasma membrane. In contrast, a surrogate OR, the GPCR mouse β2-adrenergic-receptor (mβ2AR), robustly traffics to the plasma membrane. We set out to characterize mβ2AR mutants *in vitro* for their eventual use in olfactory axon guidance studies. We performed an extensive mutational analysis of mβ2AR using a Green Fluorescent Protein-tagged mβ2AR (mβ2AR::GFP) to easily assess the extent of its plasma membrane localization. In order to characterize mutants for their ability to successfully transduce ligand-initiated signal cascades, we determined the half maximal effective concentrations (EC50) and maximal response to isoprenaline, a known mβ2AR agonist. Our analysis reveals that removal of amino terminal (Nt) N-glycosylation sites and the carboxy terminal (Ct) palmitoylation site of mβ2AR do not affect its plasma membrane localization. By contrast, when both the Nt and Ct of mβ2AR are replaced with those of M71 OR, plasma membrane trafficking is impaired. We further analyze three mβ2AR mutants (RDY, E268A, and C327R) used in olfactory axon guidance studies and are able to decorrelate their plasma membrane trafficking with their capacity to respond to isoprenaline. A deletion of the Ct prevents proper trafficking and abolishes activity, but plasma membrane trafficking can be selectively rescued by a Tyrosine to Alanine mutation in the highly conserved GPCR motif NPxxY. This new loss-of-function mutant argues for a model in which residues located at the end of transmembrane domain 7 can act as a retention signal when unmasked. Additionally, to our surprise, amongst our set of mutations only Ct mutations appear to lower mβ2AR EC50s revealing their critical role in G-protein coupling. We propose that an interaction between the Nt and Ct is necessary for proper folding and/or transport of GPCRs.

## Introduction

G-Protein Coupled Receptors (GPCRs) are the largest and most ubiquitous family of plasma membrane receptors. They are involved in almost all physiological processes in humans, such as the sensory systems of vision, taste and smell [1]. GPCRs are the most common targets of therapeutic drugs, including blockers and agonists of α and β-adrenergic receptors, dopamine receptors, serotonin receptors, histamine receptors and opioid receptors. Twenty-five percent of the top 200 therapeutics and over fifty percent of all therapeutics, target GPCRs [2]. By contrast, thousands of odorant receptors have been identified, but odor to receptor pairings have not been characterized, thus limiting the study of olfaction.

The GPCRs rhodopsin and the β2-adrenergic-receptor (β2AR) have been crystallized and extensively studied at the biochemical level, yet the vast majority of GPCR structures are undefined and their ligands remain poorly understood or unknown [3]. This is mainly due to the lack of an effective heterologous cell culture system to express GPCRs and perform high-throughput ligand binding analysis [4]. GPCRs that do not functionally express *in vitro*, fail to traffic to the plasma membrane and remain trapped in intracellular compartments [5, 6]. In contrast, some GPCRs successfully traffic to the plasma membrane of heterologous cells, like the canonical β2AR. The properties of GPCRs that govern trafficking to the plasma membrane are still largely unknown and mutants are rarely tested *in vivo*. Ironically, because the β2AR robustly traffics to the plasma membrane, its synthesis and insertion into the plasma membrane has not been the subject of experimentation. Most efforts have primarily focused on the dynamics of β2AR ligand activation.

Remarkably, when expressed from an OR locus in mouse olfactory neurons, the mouse β2AR (mβ2AR) can substitute for all functions of an OR: preventing expression of another OR, maturation of the neurons, axonal outgrowth and convergence of the axons into an homogenous ectopic glomerulus in the olfactory bulb [7]. In addition, human β2AR (hβ2AR) expressed in mouse olfactory neurons also fulfills all functions of ORs [8–10]. Hence mouse and human β2ARs can be used as OR surrogates.

To further characterize the properties of surrogate OR β2AR, we have conducted a mutational analysis to determine the features necessary for trafficking to the plasma membrane and β2AR functionality. Because β2AR normally traffics to the plasma membrane in heterologous cells, we worked backwards to determine what sequences were necessary for β2AR to function and what sequences on the non-trafficking M71 OR might “kill β2AR” trafficking and function. In a parallel study, we have also tried to “fix M71” using the opposite strategy of imparting functionality from β2AR sequences onto M71 OR [11].

Previous studies have successfully used chimeric GPCRs of the homologous receptors α2 and β2AR to identify domains involved in coupling to G-proteins and ligand binding [12], but this strategy has not been implemented to define domains critical for plasma membrane trafficking. Other groups have used chimeric analyses to rescue non-trafficking GPCRs with trafficking GPCRs, but here we are taking the reciprocal approach by replacing domains of a trafficking GPCR with domains of a non-trafficking GPCR [5].

**Fig 1:**
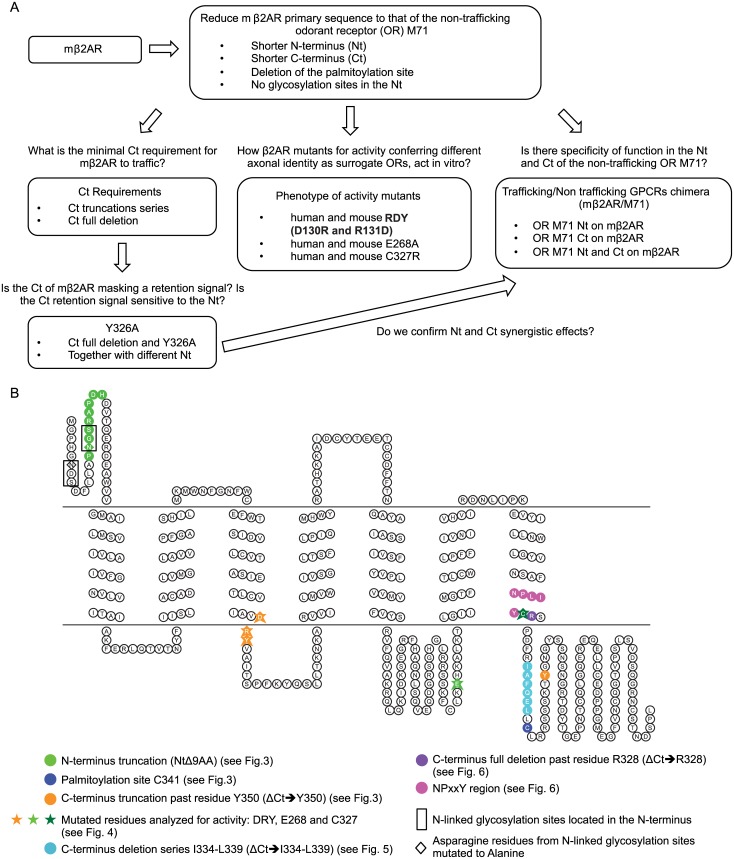
Mutational analysis for the β2 adrenergic receptor. (A) Workflow schematic of the mutational analysis for the β2 adrenergic receptor. First step was to reduce the primary sequence of the mouse β2AR (mβ2AR) to that of the non-trafficking odorant receptor M71. Then we asked what is the C-terminus minimal requirement for trafficking and activity by analyzing Ct truncations series as well as Ct full deletion. Furthermore we analyzed the effects of mutating a putative retention signal that might be revealed by a Ct deletion. We also compared the phenotype of mouse and human β2AR activity mutants to M71. Finally, we analyzed the behavior of M71/mβ2AR chimera. (B) The 2D topology of mouse β2AR was generated using the online software Topo2 (see Material & Methods). Plasma membrane is symbolized by two horizontal lines with the extracellular compartment on top and intracellular compartment on the bottom. Putative transmembrane domains are located in between those two lines. Different shapes and colors are used to symbolize the residues targeted for mutations. N-linked glycosylation sites are indicated in black boxes and their N in diamonds.

We performed a mutational analysis to test the role of N-linked glycosylation of the β2AR amino-terminus (Nt) on its trafficking capacity; we modified mβ2AR so that its primary sequence is analogous to a non-trafficking GPCR, the M71 OR, to test if the length of the Nt and/or Ct were factors involved in trafficking; we defined the role of the minimal Ct and chimeras with M71 OR on proper β2AR trafficking and activity. Finally, we analyzed known mβ2AR activity mutants on their *in vitro* trafficking defects. Over 30 mutants were analyzed. The workflow of this mutagenesis is summarized in Fig 1A.

Our analysis has identified key elements for plasma membrane trafficking and function for the mβ2AR that are potentially obscured in other non-trafficking GPCRs. Targeting these features necessary for GPCR functionality could help generate new tools for the study of olfactory function, and unravel new potential targets for therapeutics.

## Material and Methods

### Plasmid constructions

Mutant versions of human β2AR, mouse β2AR and M71 (Olfr151, codon optimized) were generated either by PCR with ultramer primers (Integrated DNA Technologies), QuickChange mutagenesis (Agilent Technologies) or Gibson Assembly Cloning (New England Biolabs Inc). See supplementary materials for DNA and protein sequences of mouse β2AR, human β2AR, M71, linker, GFP and mCherry (S1 Text). All constructs were verified through sequencing for the GPCR sequence, linker and proper phase with the fluorophore sequence.

2D topology of mouse β2AR and M71 were generated using the online software Topo2 (Johns S.J., TOPO2, Transmembrane protein display software, http://www.sacs.ucsf.edu/TOPO2/). The following putative transmembrane domain locations were used for mouse β2AR: TM1 35–58, TM2 72–95, TM3 107–130, TM4 151–174, TM5 197–220, TM6 275–298 and TM7 306–329; and the following for the mouse M71: TM1 29–49, TM2 57–77, TM3 91–111, TM4 134–154, TM5 196–216, TM6 239–259 and TM7 271–291.

RTP1S was amplified from mouse olfactory cDNA with 5’ aaaagagctcaagcttcgaattcggcgcgccaccatgtgtaagagtgtga 3’ and 5’ ttaattaatcagacagaagtacggaaggagaat 3’ primers, shuttled into the pGemT Easy vector (Promega). RTP1S clone was only used for the trafficking experiments in OP6 cells.

### OP6 cell culture and transfection

Olfactory placode 6 (OP6 [13], a gift from Jane Roskams) cells were grown in DMEM (Invitrogen), supplemented with 10% fetal bovine serum (FBS) and 1% penicillin/streptomycin, at 33°C in a humidified 5% CO2 atmosphere and passaged at regular intervals, after dissociation with trypsin 0.05%-EDTA. Cells were transiently transfected with 10μg of DNA using Amaxa Nucleofector technology (Lonza, program COS-7 DSMZ) and plated onto 60mm round dishes. Twenty-four hours after transfection, cells were imaged directly with an immersion 25X lens and a Zeiss 510 laser scanning confocal microscope.

### GFP-labeled filopodia counts

For each GFP fusion construct, 10 cells were randomly selected and GFP-labeled filopodia were counted. Confocal pictures of cells were calibrated to non-saturating images of mβ2AR::GFP. These settings were used for all subsequent images. Average and standard deviation were calculated. To assess the difference in mean filopodia counts across all samples, one-way analysis of variance (ANOVA) was performed using the MATLAB anova1 function, with an alpha = 0.001 and Scheffe post hoc tests. Mutants were categorized into groups based on their function as described in figures and ANOVA was run on each group with the addition of the M71::GFP construct as a negative control. Thus, error rates are a function of grouping.

### CellMask plasma membrane staining

Cellmask Deep Red Plasma Membrane reagent (Life Technologies) was briefly centrifuged and diluted 1/1000 in FluoroBrite DMEM (Life Technologies). Twenty-four hours after transfection (see above) and prior to imaging, cells were washed once with FluoroBrite DMEM. Cells were then stained with the diluted reagent for 7 minutes in the incubator at 33°C. After 7 minutes, cells were washed 3 times in FluoroBrite DMEM and imaged directly using a 25X immersion lens and a Zeiss LSM 510 confocal microscope.

### Dose Response Curves to isoprenaline

Calcium-based Fluorometric Imaging Plate Reader (FLIPR) experiments were performed as described previously [14]. Mutants analyzed in each figure come from the same FLIPR experiment. Stock transfection and isoprenaline solutions were prepared for each experiment to ensure the same quantity of human G-alpha15 expression and dilution of isoprenaline. Relative Fluorescent Units were baseline corrected by subtracting the lowest fluorescent measure for each experiment and a curve was fitted using a 3 parameters model (Hill Slope = 1) using the GraphPad software. Half maximal effective concentrations (EC50) were then calculated and are given in nM. The maximum response, as a percent of wild type, was calculated by reporting the span for a mutant over the span for the wild type.

## Results

### Plasma membrane localization of gap::GFP measured by GFP-labeled filopodia counts

To assess plasma membrane trafficking of odorant receptors, and other GPCRs, we have employed OP6 cells that are derived from the mouse olfactory placode [13, 15, 16]. These neuronal derived immortal cell lines naturally exhibit many filopodia—thin extensions (0.1–0.3μm diameter) from the plasma membrane mostly composed of actin filaments [17, 18]. The reduced cytoplasm fraction creates a differential between diffusion-limited cytoplasmic proteins and other protein types capable of localizing within filopodia. Indeed we previously showed that untagged GFP and six other fluorescent protein variants (XFPs) rarely enter filopodia [14].

**Fig 2:**
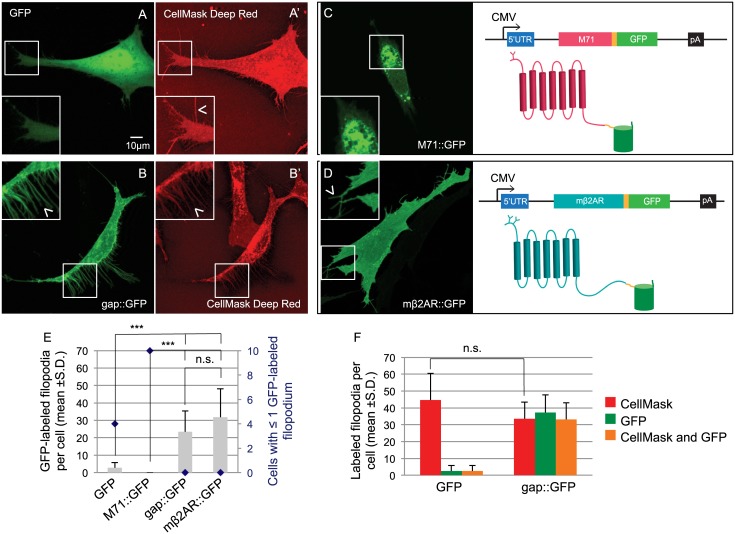
Expression of GFP and GPCR::GFP in OP6 cells. (A) Cytosolic GFP shows diffuse fluorescence in the cytoplasm of an OP6 cell and does not co-localize with the plasma membrane staining CellMask Deep Red in filopodia (A’, arrow head in magnified image). (B) On the contrary, gap::GFP, shows a sharp edge staining of a whole OP6 cell and co-localize with the plasma membrane staining CellMask Deep Red in filopodia (B’, arrow head in magnified image). (C) The M71 coding sequence missing a stop codon (pink box) was cloned followed by a linker sequence (yellow box) and the coding sequence of GFP (green box). The peGFP-N1 backbone includes the cytomegalovirus promoter (CMV, arrow), a 5’ untranslated region (5’UTR, blue box) and a polyadenylation sequence (pA, black box). The resulting 2D structure of the fusion protein is showed, the M71 odorant receptor has a putative 7 transmembrane domains structure and is glycosylated at its N-terminus (pink diagram). The GFP protein, composed of eleven β-barrels, is fused to the Ct of M71 (green diagram) after a 9 amino acids linker (in yellow). M71::GFP shows intense perinuclear localization in an OP6 cell (see picture) and does not localize to filopodia. (D) Using the same strategy the mβ2AR sequence (teal box) was cloned in the same backbone. mβ2AR has also 7 transmembrane domains but 2 N-linked glycosylation sites located in its Nt (see 2D topology). When express in OP6 cells (see picture) mβ2AR::GFP stains homogeneously the whole cell and locates in filopodia (arrow head in magnified image), like gap::GFP. (E) Cells expressing GFP and M71::GFP have very low number of GFP-labeled filopodia (gray bars, 2.8 ±2.9 and 0.0 ±0.0 GFP-labeled filopodia per cell respectively), whereas cells expressing gap::GFP and mβ2AR::GFP show the same numerous number of filopodia. In addition many cells expressing GFP and M71::GFP show ≤1 GFP-labeled filopodium whereas all cells expressing gap::GFP or mβ2AR::GFP have more filopodia (blue diamonds, 2^nd^ y axis in graph). (F) Counts of GFP- and CellMask Deep Red-labeled filopodia for cells expressing GFP or gap::GFP. The number of filopodia revealed by plasma membrane staining is the same for both type of cells (44.7 ±15.9 and 33.6 ±9.9 respectively), only in cells expressing gap::GFP-filopodia are also GFP-labeled (orange bars in graph, 2.6 ±3.2 and 33.2 ±9.8 co-labeled filopodia per cell respectively). All filopodia counts are represented as average for 10 cells ± standard deviation. ***means significantly different from mβ2AR::GFP, one-way ANOVA followed by Scheffe tests, p<0.001. n.s. means not significantly different from mβ2AR::GFP, p>0.001.

To further characterize this differential localization we compared the total number of filopodia that can be observed in a cell with the number of GFP-labeled filopodia. We show that untagged GFP fluorescence (Fig 2A) is found only in a few filopodia per cell expressing cytosolic GFP (Fig 2E). We further quantified this feature by determining how many cells show a complete absence or a single GFP-labeled filopodia (≤1). In this latter analysis, untagged GFP was observed in ≤1 filopodia in 4/10 cells (blue diamond in Fig 2E). By contrast, with simultaneous staining of the plasma membrane, using the CellMask Deep Red plasma membrane stain, we observe 44.7 ±15.9 filopodia per cell, confirming their presence, but only 2.6 ±3.2 GFP-labeled filopodia (Fig 2A’ and 2F). Thus, the lack of observable GFP-labeled filopodia in cells expressing GFP is a consequence of the inability of untagged GFP to contribute to its cytoplasmic fraction.

In contrast, when GFP is fused to a membrane targeting tag that contains a palmitoylation site from the Zebrafish Gap43 (20 amino acids, gap::GFP), it shows sharp edge expression on all cells (Fig 2B) and we observe many GFP-labeled filopodia per cell (Fig 2E). None of these cells have ≤1 GFP-labeled filopodia (Fig 2E). When this experiment is repeated with CellMask staining, all 33.6 ±9.9 filopodia per cell are completely co-labeled with gap::GFP (Fig 2B’ and 2F). Importantly, total CellMask filopodia counts between OP6 cells expressing untagged GFP or gap::GFP were comparable (p>0.001, ANOVA followed by the post hoc Scheffe tests, Fig 2F). Thus, filopodia localization of membrane targeted and fluorescently tagged proteins provides a suitable assay for plasma membrane localization in OP6 cells. Additionally, the measure of OP6 cells with ≤1 GFP-labeled filopodia (blue diamonds) proves to be inversely correlated to the average number of GFP-labeled filopodia.

### Differential plasma membrane trafficking for M71::GFP and mβ2AR::GFP

What is the cellular fate of ORs fused to a Ct GFP tag (OR::GFP) and expressed in OP6 cells? To answer this question, we chose the M71 OR gene (olfr151) since it has been well studied *in vivo* in gene-targeted and transgenic mice where M71::GFP traffics and responds to M71 specific odors in olfactory neurons ([7, 11]). M71::GFP expression in OP6 cells is observed perinuclearly (Fig 2C and 2E), which recapitulates the failed expression patterns observed for most ORs heterologously expressed in other cell types. Importantly, the filopodia are present, but M71::GFP cannot traffic to them. Filopodia are readily identified by CellMask in M71::GFP expressing OP6 cells comparable to the number of filopodia observed for GFP or gap::GFP (p>0.001, [11]).

Fusion of GFP to the Ct of mβ2AR, mβ2AR::GFP, reveals homogenous fluorescence throughout the whole cell including the filopodia, in the same manner as gap::GFP (Fig 2D vs. 2B). All cells robustly show GFP-labeled filopodia (Fig 2E) comparable to gap::GFP (p>0.001). Plasma membrane labeling with CellMask of OP6 cells expressing mβ2AR::GFP reveals that all 25.3 ±16.3 red filopodia per cell are double labeled with GFP fluorescence ([11]), which is similar to the number of CellMask labeled filopodia observed in cells expressing GFP, gap::GFP and M71::GFP (44.7 ±15.9, 33.6 ±9.9 and 34.3 ±14.4 respectively, p>0.001; Fig 2F and [11]). This analysis suggests that the number of filopodia in OP6 cells does not depend on the expressed protein, plasma membrane-associated (gap::GFP or mβ2AR::GFP) or otherwise (GFP or M71::GFP).

We have previously shown that the addition of fluorescent tags at the Ct of the mβ2AR does not inhibit trafficking of mβ2AR to the plasma membrane nor impair the functional response after ligand stimulation with isoprenaline, a known mβ2AR agonist [14]. Thus, we were in a position to quickly screen for proper trafficking of mβ2AR::GFP mutants by examining the presence of GFP-labeled filopodia and correlate this with isoprenaline-induced activation.

### N-terminal and C-terminal truncations of mβ2AR still traffic to the plasma membrane

We first sought to understand why the mβ2AR protein robustly traffics to the plasma membrane and the M71 OR protein does not. Using the crystal structure of human β2AR as a template, we set out to dissect the mβ2AR primary sequence and determine what changes could inhibit plasma membrane trafficking and/or function to the protein (Fig 1A and 1B). When compared to mβ2AR, the M71 OR has a shorter Nt (Nt: 28 vs. 34 amino acids, respectively), contains only one N-linked glycosylation site and has a much shorter Ct (Ct; 18 vs. 89 amino acids, respectively) not subject to palmitoylation. Our initial step to determine why mβ2AR traffics to the plasma membrane was to systematically reduce its primary sequence to that of the non-trafficking odorant receptor M71.

**Fig 3:**
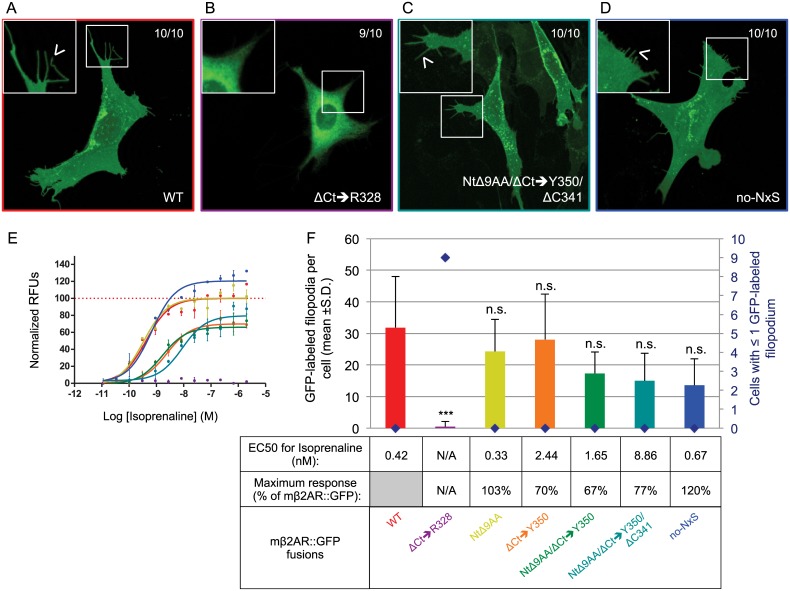
Reducing mouse β2AR primary sequence to that of the odorant receptor M71. (A) mβ2AR::GFP showing homogenous and sharp edges staining of OP6 cells (observed in 10/10 cells), is included in small isolated inclusions and also locates to filopodia (arrow head in magnified picture). (B) On the contrary, ΔCt➔R328 shows accumulation around the nucleus and does not locate to filopodia (9/10 cells, magnified picture). (C) The mutated final version of mβ2AR that looks like an OR, NtΔ9AA/ΔCt➔Y350/ΔC341, shows similar localization in OP6 cells as WT, including in filopodia (arrow head in magnified picture). (D) In the same way No-NxS shows proper trafficking in OP6 cells. (E) Dose response curves obtained by the degree of isoprenaline-induction in FLIPR experiments, are depicted as normalized relative fluorescent units (RFUs) as a function of Log [isoprenaline] (M, molar concentration). The same color code is used as in F. (F) The average number of GFP-labeled filopodia per cell is showed (bars in graph, mean +s.d.) as well as the number of cells with ≤1 GFP-labeled filopodia (blue diamonds). WT labels 31.8 ±16.2 filopodia per cell, ΔCt➔R328 0.5 ±1.6***, NtΔ9AA 24.3 ±10.2^n.s.^, ΔCt➔Y350 28.0 ±14.4^n.s.^, NtΔ9AA/ΔCt➔Y350 17.3 ±6.9^n.s.^, NtΔ9AA/ΔCt➔Y350/ΔC341 15.0 ±8.8^n.s.^, and No-NxS 13.6 ±8.3^n.s.^. ***means significantly different from WT, one-way ANOVA 8 degrees of freedom followed by Scheffe tests, p<0.001. n.s. means not significantly different from WT, p>0.001. EC50 (nM) and maximum response (expressed as a % of mβ2AR::GFP’s maximum response) for isoprenaline are indicated in the table. N/A indicates not applicable, EC50 and maximum response cannot be calculated if the mutant shows no activity in response to isoprenaline and no dose response curve can be fitted, as it is the case here for ΔCt➔R328.

First, we internally deleted the Nt of mβ2AR to be a similar length to M71, with only one N-linked glycosylation site. This 9 residues deletion, inside the Nt of mβ2AR (NtΔ9AA; green circles in 2D topology of the protein in Fig 1B), includes one of the glycosylation consensus sequences NxS. We observed normal trafficking and maximum responses to isoprenaline and the half maximal effective concentration (EC50) for NtΔ9AA comparable to WT (i.e. mβ2AR::GFP, Fig 3F and yellow curve in Fig 3E). This indicates that a shorter N-terminus with only one N-linked glycosylation site does not affect affinity of this receptor to isoprenaline or downstream ligand-induced signal transduction.

We had previously shown that the truncation of the full Ct of mβ2AR, up to and including arginine 328 (ΔCt➔R328; Fig 1B), prevents its trafficking to filopodia and response to isoprenaline [14]. Here, we also observe accumulation of the protein ΔCt➔R328 in the cytoplasm of the cells and no localization in filopodia (Fig 3B and 3F). The lack of response to isoprenaline of ΔCt➔R328 is either due to the protein not targeting to the plasma membrane, not folding properly on the way to the plasma membrane, or very low levels of protein on the plasma membrane (Fig 3E). Thus, it seemed likely that properties within the Ct are necessary for mβ2AR::GFP efficient trafficking to the plasma membrane and isoprenaline-mediated activation.

Can mβ2AR traffic and function with a short Ct? We truncated the Ct of mβ2AR up to and including residue tyrosine 350 (ΔCt➔Y350, Fig 1B), so that it contains a Ct with the same length as the M71 Ct. Plasma membrane trafficking for ΔCt➔Y350 is comparable to WT (Fig 3F). However, this Ct mutant possesses a log higher EC50 (Fig 3F) and a lower maximum response (70% of WT, Fig 3E and 3F).

Next, we truncated both the Nt and Ct of the receptor (NtΔ9AA/ΔCt➔Y350), resulting in a mβ2AR mutant with the same approximate length as M71 (341 amino acids instead of 418; -9 at Nt and -68 at Ct). Again, this fusion protein retained the capacity to traffic to filopodia (Fig 3F) and the same response profile to isoprenaline as ΔCt➔Y350 (table in Fig 3E and 3F).

Within the Ct of the human β2AR, palmitic acid was shown to bind a cysteine residue at position 341 [19]. Residue C341 is conserved in the mouse β2AR Ct (Fig 1B) but it is absent in the Ct of the non-trafficking M71 OR. Therefore, we deleted C341 in mβ2AR. The resulting mutant NtΔ9AA/ ΔCt➔Y350/ΔC341 shows normal plasma membrane trafficking and the anticipated reduced EC50 due to the ΔCt➔Y350 mutation (Fig 3C, 3E and 3F). These results show that a mutated mβ2AR that is similar in length and character to an OR, i.e., with shorter Nt and Ct, one N-linked glycosylation site in its Nt and no palmitoylation site, is still able to traffic to the plasma membrane and that these features are not essential for GPCR trafficking to the plasma membrane.

### mβ2AR N-linked glycosylation sites are not required for functionality

The NxS/T consensus sequence marks proteins for asparagine (N)-linked glycosylation. Multiple N-glycosylation sites at the Nt of GPCRs are common and thought to be involved in plasma membrane trafficking, but its role may vary among GPCRs [20]. To further explore the role of N-linked glycosylation in the Nt of mβ2AR, we mutated the only two N-linked glycosylation sites present on the extracellular domain of the entire full-length protein. We replaced the asparagine of NDS and NGS with alanine (N6A and N15A) to create no-NxS (Fig 1B). Surprisingly, this mutant showed a similar expression pattern to WT (Fig 3D).

Interestingly, the no-NxS mutant shows an increased response to isoprenaline (Maximum response is 120% of the mβ2AR::GFP response, Fig 3E and 3F) with an EC50 for isoprenaline similar to WT (0.67nM vs. 0.42nM, respectively). This suggests that the lack of Nt N-linked glycosylations in a GPCR leads to an enhanced capacity to transduce a signal in response to its ligand but is not essential for trafficking to the plasma membrane.

### Membrane localization and function of β2AR Activity Mutants

Our experiments have revealed three phenotypes: 1) membrane trafficking with EC50s similar to WT 2) membrane trafficking with higher EC50s and 3) non-membrane trafficking with absent EC50s. We sought to explore if previously characterized activity mutants of human β2AR, which had been expressed in olfactory neurons and shown to provide axons with a different identity compared to human β2AR [8], might reveal a new phenotype in our assay: one with a loss-of-function mutation (RDY) and two mutations associated with increased basal activity (E268A and C327R). We postulate that differences in their ability to traffic to the plasma membrane might also account for shown differences in axonal identity.

**Fig 4:**
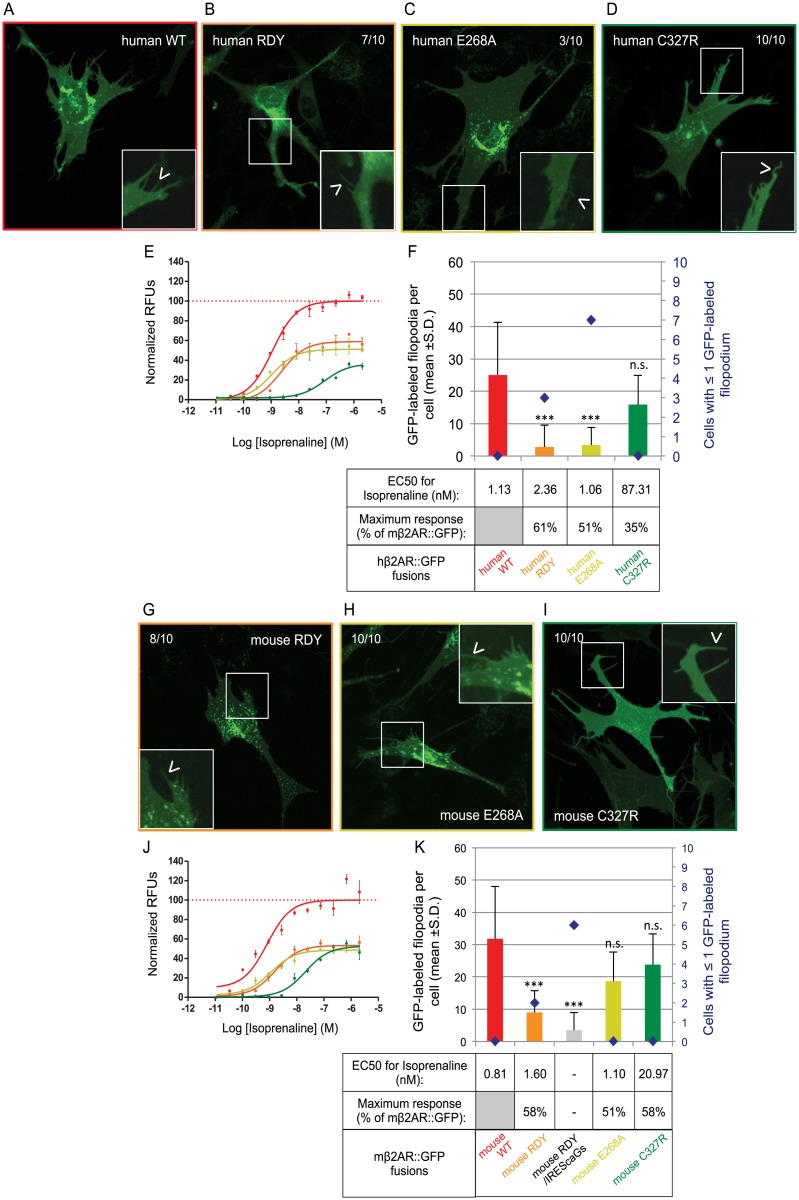
Plasma membrane trafficking of human and mouse β2AR mutants for activity. (A) The human version of β2AR::GFP shows an homogenous staining in OP6 cells, similar in localization to mβ2AR::GFP, but more faint (observed in 10/10 cells). The protein also locates to small isolated inclusions and filopodia (arrow head in magnified picture), also at a lower level than mβ2AR::GFP. In this figure all zoom-in pictures are corrected for contrast to allow good display of filopodia. (B) On the contrary, the activity mutant human RDY shows accumulation in the periphery of the nucleus and locate in fewer filopodia (7/10 cells, arrow head in magnified picture). (C) The human E268A protein shows uniform and faint staining of the cell in addition to small inclusions, but rarely locates to filopodia (only in 3/10 cells, arrow head in magnified picture). (D) Human C327R shows similar localization than human WT and locates to filopodia (10/10 cells, arrow head in magnified picture). (E) Dose response curves to isoprenaline obtained by FLIPR analysis, are showed as normalized relative fluorescent units (RFUs) as a function of Log [isoprenaline] (M). The same color code is used as in F. (F) The average number of GFP-labeled filopodia per cell is showed (bars in graph, mean +s.d.) as well as the number of cells with ≤1 GFP-labeled filopodia (blue diamonds). Human WT labels 25.0 ±10.7 filopodia per cell, human RDY only 2.8 ±1.7***, human E268A 3.4 ±7.3*** and human C327R 15.9 ±9.7^n.s.^. ***means significantly different from hβ2AR::GFP, one-way ANOVA 3 degrees of freedom followed by Scheffe tests, p<0.001. n.s. means not significantly different from hβ2AR::GFP, p>0.001. EC50 (nM) and maximum response (expressed as a % of hβ2AR::GFP’s maximum response) for isoprenaline are indicated in the table. (G) As the human version, mouse RDY shows normal localization inside the cell (8/10 cells) but traffic to only few filopodia (arrow head in magnified picture). (H) However, mouse E268A does not behave like human E268A, it shows normal localization inside the cell (10/10 cells) and in filopodia (arrow head in magnified picture). (I) Mouse C327R, as the human version and as WT, shows homogenous staining of the cell and few inclusions (10/10 cells) and locates to filopodia (arrow head in magnified picture). (J) Dose response curves to isoprenaline obtained by FLIPR analysis, are showed as normalized relative fluorescent units (RFUs) as a function of Log [isoprenaline] (M). The same color code is used as in K. (K) The average number of GFP-labeled filopodia per cell is showed (bars in graph, mean +s.d.) as well as the number of cells with ≤1 GFP-labeled filopodium (blue diamonds). Mouse WT labels 31.8 ±16.2 filopodia per cell, mouse RDY 9.0 ±6.7***, mouse RDY/IREScaGs 3.5 ±5.5***, mouse E268A 18.7 ±9.0^n.s.^ and mouse C327R 23.8 ±9.5^n.s.^. ***means significantly different from WT, one-way ANOVA 4 degrees of freedom followed by Scheffe tests, p<0.001. n.s. means not significantly different from WT, p>0.001. EC50 (nM) and maximum response (expressed as a % of mβ2AR::GFP’s maximum response) for isoprenaline are indicated in the table. Mouse RDY/IREScaGs was not tested for activity.

A highly conserved region aspartic acid-asparagine (DRY) suspected to be involved in G-protein coupling has been previously mutated to asparagine-aspartic acid (RDY) in the rat I7 (rI7) OR and was non-functional. Neurons expressing rI7(RDY) do not show Ca^2+^ signals in response to its cognate ligand, octanal, and their axons fail to develop normal connections [21, 22]. Like many other ORs, the rI7(RDY) protein is unlikely to traffic to the plasma membrane in heterologous cells [23] and its trafficking has not been analyzed in olfactory neurons. Thus, loss-of-function in signaling and axonal phenotypes for the RDY mutant could also be explained by a loss of membrane trafficking. In order to analyze the effect of the RDY mutation on plasma membrane trafficking we generated the same mutations in the conserved region in both mouse and human β2AR (Fig 1B). Surprisingly, we observe isoprenaline responses for both mouse and human RDY mutants (Fig 4E, 4F, 4J and 4K), with a maximum response rate of ~60% when compared to WT for both mutants. However, their EC50s are two-fold higher when compared to their WT counterparts. Both mouse and human RDY mutants traffic poorly to the plasma membrane and GFP-labeled filopodia are significantly lower in number. The human RDY mutant shows some accumulation in the periphery of the nucleus (Fig 4B), whereas the mouse RDY mutant accumulates protein in punctate inclusions within the cells and shows relatively poor expression in the rest of the cell (Fig 4G). In summary, our results reveal that the RDY mutation impairs both ligand-induced activity and plasma membrane trafficking for the β2AR.

In olfactory neurons, co-expression (using an Internal Ribosome Entry Site or IRES sequence) of a constitutively active form of a G-protein (caGs), together with rI7(RDY), rescues some of the receptor function in axonal guidance [21]. Since G-proteins are known associates of GPCRs, it is possible that caGs enhance trafficking of an RDY mutant. This prompted us to determine if the filopodia localization of the mouse RDY mutant is affected when co-expressed with caGs (mouse RDY/IREScaGs). Co-expression of caGs did not rescue plasma membrane trafficking of mouse RDY (Fig 4K). This suggests that the rescue of the axonal targeting phenotype observed *in vivo* for rI7(RDY)-IRES-caGs is not due to a change in plasma membrane trafficking of the receptor.

Subsequently, we tested two mutations with increased basal activity (E268A and C327R; Fig 1B) in both human and mouse β2AR. The charge-neutralizing mutation E268A has been previously described to increase both basal and induced activity of hβ2AR (increase of cyclic AMP accumulation in transiently transfected COS-7 cells) [24]. In addition, the mutation reduced the expression level in cells, possibly due to an increased instability of the protein [24]. In our paradigm, we did not observe an increase of response to isoprenaline for both the human and the mouse E268A mutant. On the contrary, the maximal isoprenaline responses for both are reduced (Mouse: Fig 4E and 4F, Human: Fig 4J and 4K), but their respective EC50s are similar to their WT counterparts (Fig 4F and 4K). Strangely, the plasma membrane trafficking of mouse E268A appears normal (Fig 4H and 4K) whereas the human E268A fails to traffic properly to filopodia (Fig 4C and 4F). This is consistent with data from Ballesteros et al. [24] suggesting a reduced level of expression of the human activity mutant. We conclude that the E268A mutation is a partial loss-of-function mutant rather than a gain-of-function, as was previously described.

Thereafter, we analyzed the plasma membrane expression for the C327R mutation in both the human and mouse β2AR (Fig 1B), which was previously described to reduce the binding affinity of hβ2AR for isoprenaline and the efficacy of the receptor to stimulate adenylyl cyclase [25]. We confirm that both human C327R and mouse C327R mutants show much higher EC50s to isoprenaline (87.31nM and 20.97nM, respectively) and decreased maximum responses (35% and 58%, respectively) when compared to their WT counterparts (Fig 4E, 4F, 4J and 4K). Unlike RDY mutations and the mouse E268A mutation, both the human and mouse C327R mutants traffic well to the plasma membrane (Fig 4D, 4F, 4I and 4K).

Examination of these three altered-function activity mutants reveals two additional phenotypes: 1) membrane trafficking with ≥1 log higher EC50s and 2) poor membrane trafficking with ≥1 log higher EC50s. Moreover, the human and mouse β2ARs appear to be differentially affected in their trafficking for the same mutation.

### A minimal C-terminus sequence requirement for mβ2AR

**Fig 5:**
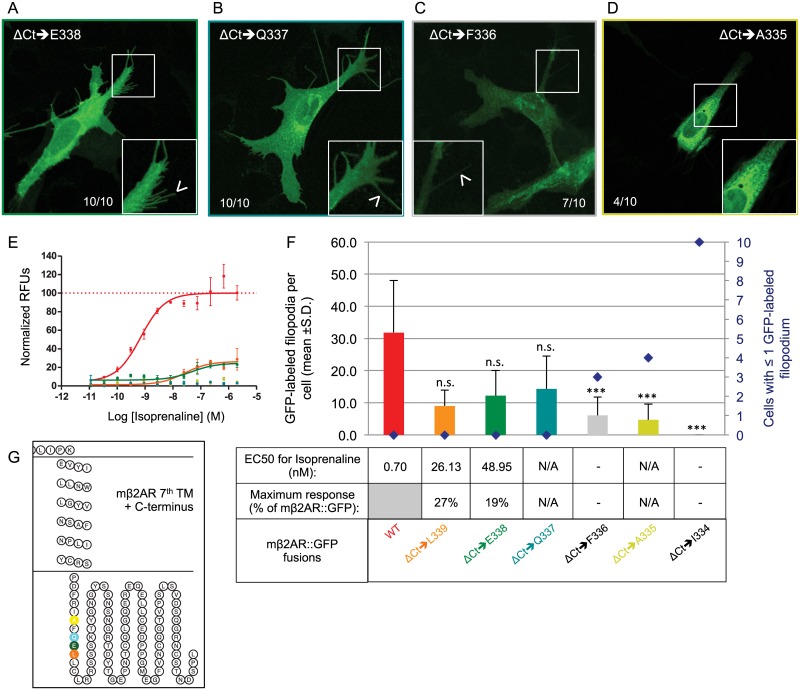
Minimal Ct requirements for mouse β2AR plasma membrane trafficking and activity. (A and B) Truncation of the Ct of mβ2AR to E338 and Q337 does not impair proper trafficking of the protein in the cell (both 10/10 cells) and ΔCt➔E338 and ΔCt➔Q337 successfully traffics to filopodia in both cases (arrow head in magnified pictures). (C and D) On the contrary, truncated proteins to F336 and A335 show accumulation at the margin of the nucleus and very rarely locate to filopodia (arrow head in magnified pictures). (E) Dose response curves to isoprenaline obtained by FLIPR analysis, are showed as normalized relative fluorescent units (RFUs) as a function of Log [isoprenaline] (M). The same color code is used as in F. (F) The average number of GFP-labeled filopodia per cell is showed (bars in graph, mean +s.d.) as well as the number of cells with ≤1 GFP-labeled filopodium (blue diamonds). ΔCt➔L339 labels 9.0 ±4.9^n.s.^ filopodia per cell, ΔCt➔E338 12.2 ±7.9 ^n.s.^, ΔCt➔Q337 14.3 ±10.2 ^n.s.^, ΔCt➔F336 6.0 ±5.7***, ΔCt➔A335 13.5 ±10.1*** and ΔCt➔I334 0.0 ±0.0***. ***means significantly different from WT, one-way ANOVA 6 degrees of freedom followed by Scheffe tests, p<0.001. n.s. means not significantly different from WT, p>0.001. EC50 (nM) and maximum response (expressed as a % of mβ2AR::GFP’s maximum response) for isoprenaline are indicated in the table. (G) Snake plot of the 7^th^ and last transmembrane domain and the Ct of the protein showing the localization of the last residues included in the truncation series.

In order to determine the critical role of the Ct for mβ2AR plasma membrane trafficking, we performed a series of C-terminal truncations (Fig 1B). The phenotype of the ΔCt➔R328 mutation that we previously characterized is slightly unexpected as the mβ2AR protein is linked to the Ct GFP tag via a Ct 9 amino acid linker (LINDPPVAT), both of which could conceivably replace the 21 amino acids that were deleted between R328 and Y350 (plus ΔC341). Because ΔC341 did not alter localization of mβ2AR to the plasma membrane we postulate that amino acids between L340 and R328 are critical. Thus, we stepwise deleted amino acids to create the mutant series ΔCt➔L340 to ΔCt➔I334. We observe that mutants with a Ct truncated up to and including leucine 340 (ΔCt➔L340, data not shown), leucine 339 (ΔCt➔L339), glutamic acid 338 (ΔCt➔E338), and glutamine 337 (ΔCt➔Q337), traffic effectively to filopodia (Fig 5A, 5B and 5F). In contrast, mutants with a truncation up to and including phenylalanine 336 (ΔCt➔F336), alanine 335 (ΔCt➔A335) or isoleucine 334 (ΔCt➔I334), fail to traffic successfully to filopodia (Fig 5C, 5D and 5F).

One interpretation of our deletion series is that a Ct tail of 8 amino acids (PDFRIAFQ) is necessary for the receptor to traffic successfully to filopodia. Normally the Ct associates with the plasma membrane through the palmitoylation of the C341 residue. Thus, the 13 residues from 328 to 341 might have a specific hydrophobic character to allow this event to occur and a change in this overall hydropathic character might explain why different versions of the Ct do not allow for trafficking. The 8 amino acids sequence PDFRIAFQ has a composite hydropathy index of -2.0 [26]. Removal of Q337 (the last amino acid needed for trafficking) alters the composite hydropathy index to a net positive of 1.5. The 9 amino acids, LINDPPVAT, in the GFP-linker sequence also have a net positive composite hydropathy index of 3.4 and perhaps it explains why it does not substitute. However, the change in charge cannot be the sole reason explaining the obstruction of trafficking, as the additional loss of F336 and F336+A335 alter the hydropathy to a net negative charge of the remaining 8 or 7 residues, but do not rescue trafficking.

We have previously observed that ΔCt➔R328 fails to traffic and fails to elicit isoprenaline responses. These two qualities need not be correlated. Indeed, when we test ΔCt truncation mutants for their responses to isoprenaline, we do not find isoprenaline-mediated responses for ΔCt➔Q337 (Fig 5E) and ΔCt➔A335 (Fig 5E), despite ΔCt➔Q337 trafficking to filopodia (see above). The observed membrane trafficking for ΔCt➔L339 and ΔCt➔E338 (Fig 5E) correlates to only small isoprenaline responses (Fig 5F, maximum responses are 27% and 19% of WT respectively and their EC50s are 2 logs higher than the WT). Thus, the first nine residues of mβ2AR Ct are needed to initiate even small isoprenaline responses.

### Phenotypic rescue of C-terminus requirement

**Fig 6:**
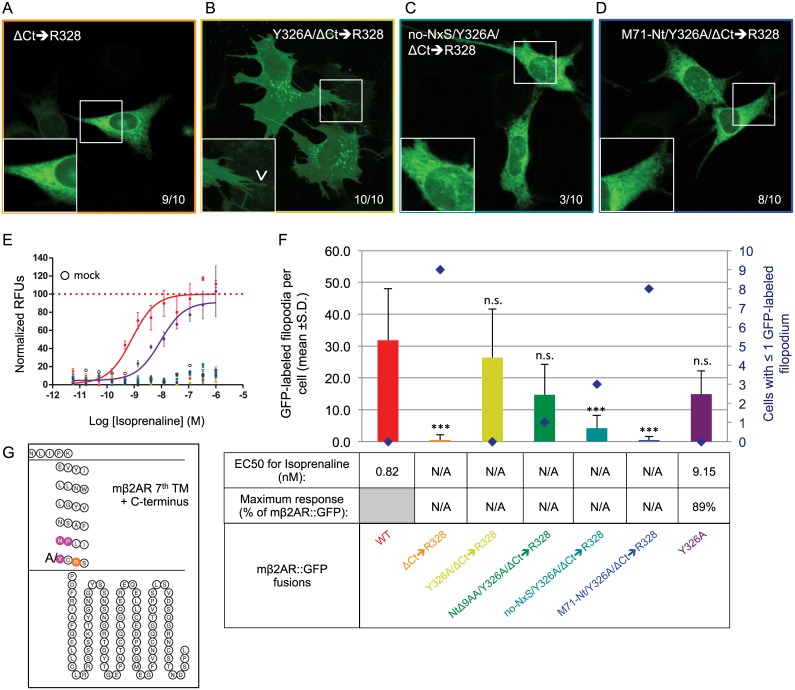
A new loss-of-function mutants for activity which retains trafficking. (A) A complete deletion of the Ct of mβ2AR to R328 blocks proper trafficking of the protein, which appears to be retained in the periphery of the nucleus (ΔCt➔R328, 9/10 cells) and does not traffic to filopodia (magnified pictures). (B) The mutation Y326A rescues trafficking of the protein in the cell (Y326A/ΔCt➔R328, 10/10 cells) and to filopodia (arrow head in magnified pictures). (C and D) This rescue is not observed anymore when the 2 N-linked glycosylation sites located in the Nt are invalidated (no NxS / Y326A/ΔCt➔R328) or when the Nt is replaced by the one of M71 (M71 Nt / Y326A/ΔCt➔R328) (respectively 3/10 cells and 8/10 cells). In both cases the protein fails to traffic to filopodia (magnified pictures). (E) Dose response curves to isoprenaline obtained by FLIPR analysis are showed as normalized relative fluorescent units (RFUs) as a function of Log [isoprenaline] (M). The same color code is used as in F. (F) The average number of GFP-labeled filopodia per cell is showed (bars in graph, mean +s.d.) as well as the number of cells with ≤1 GFP-labeled filopodium (blue diamonds). ΔCt➔R328 labels only 0.5 ±1.6*** filopodia per cell, whereas Y326A/ΔCt➔R328 26.4 ±15.2 ^n.s.^, NtΔ9AA/Y326A/ΔCt➔R328 14.7 ±9.5 ^n.s.^, no NxS / Y326A/ΔCt➔R328 4.2 ±4.0***, M71 Nt / Y326A/ΔCt➔R328 0.5 ±1.1*** and Y326A 14.9 ±7.3 ^n.s.^. ***means significantly different from WT, one-way ANOVA 6 degrees of freedom followed by Scheffe tests, p<0.001. n.s. means not significantly different from WT, p>0.001. EC50 (nM) and maximum response (expressed as a % of mβ2AR::GFP’s maximum response) for isoprenaline are indicated in the table. (G) Snake plot of the 7^th^ and last transmembrane domain and the Ct of the protein showing the localization of the NPxxY region as well as R328.

Our ΔCt truncation mutants show that the mβ2AR Ct tail is involved in G-protein coupling. However, these mutants do not unravel the reason why plasma membrane trafficking fails to occur in ΔCt➔R328, ΔCt➔I334, ΔCt➔A335, and ΔCt➔F336 (Figs 3B, 3F, 6A and 6F). One possible explanation is that the protein needs a specific sequence motif in the Ct tail for proper folding, but the fusion protein still has a cytoplasmic tail from the linker and GFP tag and their sequences are not markedly different from those of mβ2AR. Another possibility is that the mβ2AR protein contains a retention signal sequence that is usually masked by the Ct tail [27]. We therefore looked for a conserved motif near the mβ2AR Ct that might act as a retention sequence. At the end of the last trans-membrane domain of mβ2AR is a highly conserved NPxxY region (NPLIY, Figs 1A and 6G). This region, highly conserved throughout the GPCR superfamily, was shown to be involved in internalization of several GPCRs to the endosomes [20]. Alanine scanning mutagenesis of the three conserved residues reveal that only the isolated substitution of Y326A alters trafficking of the protein to the membrane without the presence of a C-terminus (Neither **A**PLIY, N**A**LIY, nor **AA**LI**A** can rescue the non-trafficking phenotype). The Y326A/ΔCt➔R328 mutant shows expression similar to mouse WT (Fig 6B and 6F). Interestingly, both ΔCt➔R328 and Y326A/ΔCt➔R328 show no capacity to signal after isoprenaline exposure (Fig 6E, comparable to no plasmid-mock transfection). Thus, we have revealed a major role of the tyrosine residue in the context of the conserved region NPxxY as a retention signal, if exposed, and we describe a new loss-of-function mutant Y326A/ΔCt➔R328, which retains normal plasma membrane trafficking.

### Y326A rescue is dependent on N-terminal amino acids

We previously observed subtle differences in trafficking and maximum isoprenaline responses when Nt residues are modified (Fig 3). It is plausible that the Ct truncation mutation that does not G-protein couple, Y326A/ΔCt➔R328, might also have its plasma membrane trafficking affected by Nt modifications. A shorter Nt with a single N-linked glycosylation site, NtΔ9AA/Y326A/ΔCt➔R328, retains normal trafficking (Fig 6F and S1A Fig). Surprisingly, we do not observe trafficking when both N-linked glycosylation sites are mutated (no-NxS/Y326A/ΔCt➔R328, Fig 6C and 6F) nor when the Nt is replaced by the Nt of M71 (M71-Nt/Y326A/ΔCt➔R328, Fig 6D and 6F). All mutants with ΔCt➔R328 are not induced by isoprenaline (Fig 6E and 6F, comparable to mock). The latter two mutants suggest a synergistic interaction between the Nt of the protein and the Y326A mutation near the Ct of the protein. In addition, we reveal a glycosylation dependent plasma membrane trafficking phenotype. Curiously, the M71-Nt completely abolishes all signs of membrane trafficking in almost all fluorescent cells. Thus, the Nt and Ct interactions may play a bigger role in membrane trafficking than previously considered.

Finally, we determined if the Y326A mutation has an effect on the full length mβ2AR::GFP protein. We show that Y326A does not affect trafficking (Fig 6F and S2B Fig), but slightly reduces the maximal response to isoprenaline (89% of the mouse WT) and increases the EC50 by one log (Fig 6E and 6F). Therefore, the residue Y326 may have a role in the G-protein coupling mechanism.

### Synergistic action between the Nt and Ct of mβ2AR

**Fig 7:**
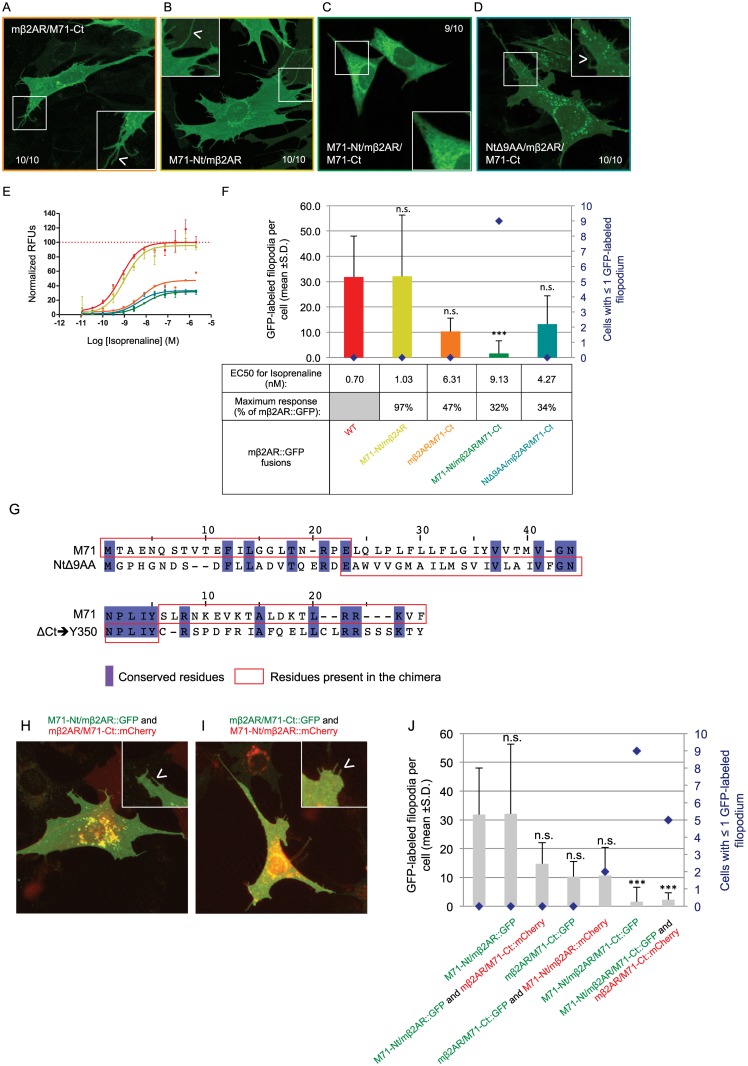
The N- and C- termini of the odorant receptor M71 impair trafficking. (A and B) The replacement of either the Ct or the Nt of mβ2AR with the one of M71 does not block proper trafficking of the protein inside the cell (both 10/10 cells) and to filopodia (arrow head in magnified pictures). (C) On the contrary the double swaps of both the Nt and Ct of mβ2AR with the Nt and Ct of M71 blocks proper trafficking of the protein, which accumulates in the periphery of the nucleus (9/10 cells) and does not locate to filopodia (magnified pictures). (D) This is not observed for a mutant with a shorter Nt and the Ct of M71 (10/10 cells) and the protein successfully traffics to the filopodia (arrow head in magnified pictures). (E) Dose response curves to isoprenaline obtained by FLIPR analysis are showed as normalized relative fluorescent units (RFUs) as a function of Log [isoprenaline] (M). The same color code is used as in F. (F) The average number of GFP-labeled filopodia per cell is showed (bars in graph, mean +s.d.) as well as the number of cells with ≤1 GFP-labeled filopodium (blue diamonds). M71-Nt/mβ2AR labels 32.1 ±24.2 ^n.s.^ filopodia per cell, mβ2AR/M71-Ct 10.3 ±5.2 ^n.s.^, M71-Nt/mβ2AR/M71-Ct 1.6 ±5.1*** and NtΔ9AA/mβ2AR/M71-Ct 13.2 ±11.3 ^n.s.^. ***means significantly different from WT, one-way ANOVA 6 degrees of freedom followed by Scheffe tests, p<0.001. n.s. means not significantly different from WT, p>0.001. EC50 (nM) and maximum response (expressed as a % of mβ2AR::GFP’s maximum response) for isoprenaline are indicated in the table. (G) Top pair is the alignment between the Nt of M71 and the truncated version of the Nt of mβ2AR (NtΔ9AA) and on the bottom pair is the alignment between the Ct of M71 and the truncated version of the Ct of mβ2AR (ΔCt➔Y350). Conserved residues are in violet and sequences present in the chimera are in the red boxes. (H) The chimera M71-Nt/mβ2AR::GFP shows normal localization in cells and localize to filopodia (arrow head in magnified picture) when co-expressed with mβ2AR/M71-Ct::mCherry. (I) mβ2AR/M71-Ct::GFP also locates as the WT in cell including to filopodia (arrow head in magnified pictures) when co-expressed with M71-Nt/mβ2AR::mCherry. (J) The average number of GFP-labeled filopodia per cell is showed (bars in graph, mean +s.d.) as well as the number of cells with ≤1 GFP-labeled filopodium (blue diamonds). M71-Nt/mβ2AR::GFP labels 32.1 ±24.2 ^n.s.^ filopodia per cell and 14.8 ±7.3 ^n.s.^ when co-expressed with mβ2AR/M71-Ct::mCherry. mβ2AR/M71-Ct::GFP labels 10.3 ±5.2 ^n.s.^ filopodia per cell and 10.7 ±9.7 ^n.s.^ when co-expressed with M71-Nt/mβ2AR::mCherry. M71-Nt/mβ2AR/M71-Ct::GFP labels only 1.6 ±5.1*** filopodia per cell and 2.2 ±2.6*** when co-expressed with mβ2AR/M71-Ct::mCherry.

Our mβ2AR, modified to resemble the non-trafficking receptor M71, NtΔ9AA/ΔCt➔Y350/ΔC341, retains the capacity to traffic to filopodia, albeit with reduced activity. This result indicates that it is not simply the length of the Nt and the Ct of the M71 OR that prevents its plasma membrane expression. Both no-NxS/Y326A/ΔCt➔R328 and M71-Nt/Y326A/ΔCt➔R328 mutants suggest that a synergistic interaction is occurring between the two ends of the mβ2AR molecule. We wanted to ask if there could be a synergistic action between the M71-Nt and M71-Ct. Therefore, we swapped the Nt and Ct of the mβ2AR with those of M71. The residues of M71-Nt to swap were defined through alignment with the shorter Nt of mβ2AR, NtΔ9AA, and the residues of M71-Ct to swap were defined through alignment with the shorter Ct version of mβ2AR, ΔCt➔Y350 (see alignments in Fig 6G). The M71-Nt/mβ2AR chimera successfully traffics to filopodia (Fig 7B and 7F) with maximal response and EC50 to isoprenaline comparable to WT as well (Fig 7E and 7F). We have shown that a short Ct tail, ΔCt➔Y350, is non-restrictive for proper trafficking of the receptor to the plasma membrane. When we analyze the mβ2AR/M71-Ct chimera, the resulting mutant appears to traffic efficiently to the plasma membrane (Fig 7A and 7F), but its maximum response to isoprenaline is only 47% of WT and its EC50 is one log higher (Fig 7E and 7F). Therefore, neither the Nt nor Ct of M71 on their own can account for the failure of M71 to traffic to the plasma membrane in heterologous cells. However, the addition of the M71-Ct does significantly reduce the response to isoprenaline, similar to what is observed for NtΔ9AA/ΔCt➔Y350/ΔC341 with analogous length of the Ct tail and missing its palmitoylation site.

Simultaneous replacement of the Nt and the Ct of mβ2AR with that of M71 reveals a synergistic affect. The M71-Nt/mβ2AR/M71-Ct mutant accumulates in the periphery of the nucleus (Fig 7C) and fails to traffic to filopodia (Fig 7F). Here, we recapitulate the synergistic effects observed in M71-Nt/Y326A/ΔCt➔R328 (Fig 6F). Despite M71-Nt/mβ2AR/M71-Ct trafficking very poorly to the plasma membrane, it maintains a maximum response that is 32% of WT and its EC50 is 1 log higher than WT (Fig 7E and 7F). When the M71-Nt is replaced with a shorter mβ2AR-Nt, resulting in NtΔ9AA/mβ2AR/M71-Ct, the trafficking deficit is corrected, but the maximal response or EC50 are not (Fig 7D and 7F). This suggests that the negative impact of M71-Nt together with M71-Ct on the same protein is not due to the shortness of the Nt but rather to some specific aspect of the M71 sequence. In addition, the maximal responses and EC50s in the context of three different mβ2AR/M71-Ct mutants do not correlate with the level of plasma membrane expression, but rather with the presence of the M71-Ct residues. These results suggest that, in our assays, very little protein is necessary at the plasma membrane to produce isoprenaline responses similar to mβ2AR mutants that show robust trafficking to the plasma membrane.

### Intra-molecule interactions required for Nt and Ct synergistic effects

To distinguish between an intramolecular Nt and Ct interaction and an intermolecular interaction between the Nt and Ct from different molecules of mβ2ARs, we tested if the observed trafficking deficits brought on by the presence of M71 Nt and Ct can be recapitulated if located on two different proteins. We co-transfected cells with the chimeras M71-Nt/mβ2AR::GFP and mβ2AR/M71-Ct::mCherry. The number of GFP-labeled filopodia observed, is comparable to both WT and M71-Nt/mβ2AR::GFP expressed alone (Fig 7H and 7J). The results are similar in the reciprocal co-transfection experiment: mβ2AR/M71-Ct::GFP with M71-Nt/mβ2AR::mCherry (Fig 7I and 7J). Thus, the Nt and Ct synergistic effects require intra-molecular interactions to block plasma membrane trafficking. This synergistic effect between the Nt and Ct may partially explain why some GPCRs, like ORs, do not traffic in heterologous cells.

## Discussion

### A new easy and robust assay for plasma membrane localization of GPCRs

We have further developed our GPCR expression-profiling assay using C-terminal GFP fusions for the rapid analysis of mutant GPCR localization throughout the cell without the need for antibody staining. Our previous analysis of mβ2AR fused to 7 different fluorescent proteins (XFPs) from four species revealed that mβ2AR::XFPs traffic to the plasma membrane and retain functionality equivalent to untagged mβ2AR. In particular, GFP appears to be nearly inert in fusion proteins [14].

We took advantage of the OP6 cell morphology, as they have abundant filopodia where GFP does not traffic unless fused to a plasma membrane protein. We observed an average of 34.5 ±15.4 filopodia (observed by CellMask staining) per OP6 cell expressing either GFP, gap::GFP, mβ2AR::GFP or M71::GFP. The number of filopodia is therefore unaffected by the type of protein expressed in this study. Quantification of GFP-labeled filopodia is an easy and robust method to test for plasma membrane localization.

Even though epitope identification can be advantageous in the detection of very low protein levels, especially at the plasma membrane, these detection schemes require live-cell antibody staining [28], without cell permeabilization, so that only the protein present at the plasma membrane can be bound by the antibody. This antibody assay reveals only a partial view of protein trafficking within the cell. For instance, some studies of non-trafficking ORs show live-cell antibody staining at the membrane, which is marginal compared to the preponderance of the protein stuck inside the cell when revealed after permeabilization [29]. Moreover an antibody recognizing an extracellular epitope is needed, restricting the number of GPCR mutants that can be analyzed. When such antibodies are not available, which is most of the time, tags (FLAG, RHO, HA and LUCY) are often added to the Nt of the recombinant protein [4]. On the contrary, fluorescent protein fusions can be added to the Ct of any GPCR or mutant of interest, allowing the visualization of both plasma membrane localization as well as any intracellular compartment localization simultaneously. In addition, we do not use Nt tags assuring that the critical initial steps of translation, endoplasmic reticulum (ER) trafficking and membrane insertion, are a representation of the cognate amino acids. Ironically, Nt tags such as FLAG, RHO, HA and LUCY are typically used to improve ER trafficking and protein expression, which is useful for heterologous cell expression in many studies but would confound our structure-function analysis.

We have efficiently begun to define the characteristics of GPCRs that traffic to the plasma membrane. Previous studies by others already analyze Ct truncations [30] or chimeric receptors [5, 31] but, in most cases, with Nt-tags. Our analysis deconstructs GPCR trafficking and G-protein coupling. For the first time this assay allows to easily perform an extensive site-directed mutagenesis of GPCRs without the use of antibodies.

The dynamic range of our GPCR::GFP-labeled filopodia averages is between 0 and 32.1 per cell. All average counts above 10 (19/19), readily described a mutant as trafficking to the plasma membrane. Only one mutant, ΔCt➔L339, appeared to traffic with a GFP-labeled filopodia average count less than 10. We also show that 19/20 trafficking mutants do not have any cells with ≤1 GFP-labeled filopodia. This inverse correlation holds true for 13/13 non-trafficking mutants tested (see blue diamonds in S3 Fig; only NtΔ9AA/Y326A/ΔCt➔R328 breaks this correlation). All non-trafficking mutants have at least 2/10 cells with ≤1 GFP-labeled filopodia. Hence, our two measures of GPCR membrane trafficking are highly correlated, reinforcing our confidence that the number of GFP-labeled filopodia for 10 cells is a sufficient measure for proper expression of a given GPCR in OP6 cells.

### N-linked glycosylations of the Nt and palmitoylation are not essential features for GPCR trafficking

Our results suggest that N-linked glycosylation sites at the Nt of a GPCR are not necessary for robust mβ2AR plasma membrane trafficking or G-protein coupling. A previous study has showed, through a ligand binding assay in COS-7 cells, that only 52% of the hamster N6Q N15Q β2AR was expressed on the cell surface, compared to 95% of the WT hamster receptor [32]. In contrast, our study reveals an increase in maximal response to isoprenaline (120% of the mouse WT) without significant change to the EC50 and trafficking. This different phenotype observed could reveal species-specific features of the receptor, as we show for activity mutants between human and mouse β2AR. Such increased activity for a GPCR glycosylation mutant has already been described, as the mutated Protease-activated receptor-1 (PAR1) lacking glycosylation of its 2^nd^ extracellular loop showed a greater efficacy in G-protein signaling [33]. Likewise, the glycosylation status of a protein can be cell-type specific, and our results could reveal that N-linked glycosylation of the N-terminus of a GPCR is not a necessary feature for proper trafficking in an olfactory placode-derived cell line or HEK293T cells (used for dose response curves). We have only characterized N-linked glycosylation mutants with the high-affinity isoprenaline ligand. It is known that a given GPCR can activate different pathways and that some ligands activate different subsets of those effectors [34], hence other ligands may depend on N-linked glycosylation sites to activate mβ2AR. We observe that the addition of various N-linked glycosylation sites to the N-terminus of the M71 OR, do not rescue protein trafficking to the plasma membrane [11]. Taken together, these results suggest that N-linked glycosylation at the N-terminus of a GPCR is neither necessary nor sufficient for membrane trafficking of the protein. This result is rather surprising, as the *in vivo* M71 OR mutation N5Q, disrupting its only potential glycosylation site, can no longer promote axon guidance, but is likely still able to lock in OR gene choice [7]. It will be interesting to see if glycosylation of mβ2AR is necessary for its ability to act as a surrogate OR *in vivo*.

Next, we showed that palmitoylation of mβ2AR is unnecessary for proper trafficking to the filopodia of OP6 cells. Binding of palmitic acid to cysteine 341 (C341) in the Ct of human β2AR allows for anchoring of the protein to the membrane. This anchoring seems not important for trafficking but rather for G-protein coupling. Indeed deleting C341 lowers the isoprenaline-induced activity by 5 fold, EC50 of 1.65nM for NtΔ9AA/ΔCt➔Y350 compared to 8.86nM for NtΔ9AA/ΔCt➔Y350/ΔC341. It is possible that signaling pathways using different G-proteins or β-arrestins have been disrupted as well.

### β2AR Activity Mutants have different trafficking phenotypes

**Fig 8:**
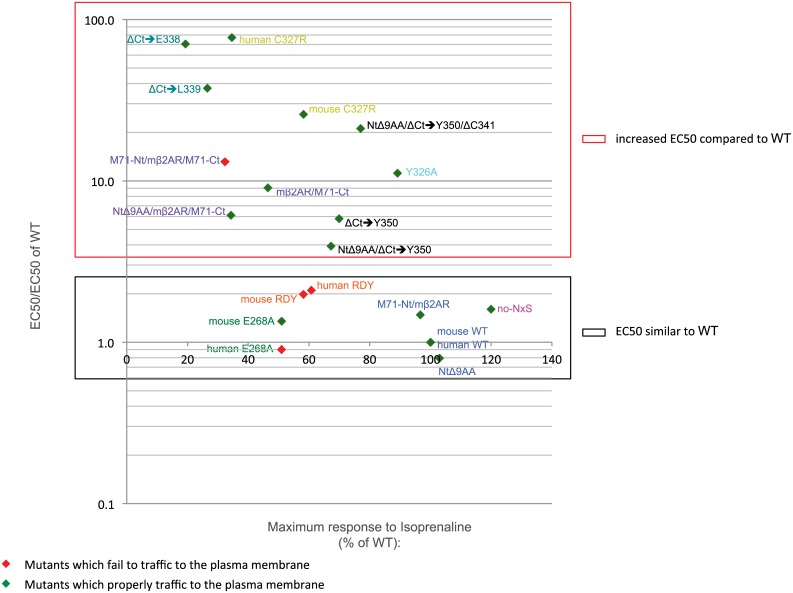
Plot for relative EC50 and % of maximum response. All mutants are positioned for their relative EC50 for isoprenaline (EC50/EC50 of WT) on the y-axis (log scale) and their maximum response as a % of WT on the x-axis. Mutants that fail to traffic to the plasma membrane are depicted as red diamonds and mutants that properly traffic to the plasma membrane are depicted as green diamonds. The red box indicates all the mutants with a lower affinity for isoprenaline than WT i.e. EC50/EC50 of WT >>1 (from 3.9 to 77.4). All these mutants have mutations in the last 7^th^ transmembrane domain or the Ct of the protein. The black box includes all the mutants that have a similar affinity for isoprenaline than WT i.e. EC50/EC50 of β2AR::GFP ≈ 1 (from 0.8 to 2.1).

The study of OR function in olfactory neurons has been limited due to our inability to test *in vitro* mutants with possible altered signaling abilities. Human β2AR mutants have been tested *in vivo* for their function in axon guidance, but have yet to be carefully characterized *in vitro* using isoprenaline-induced signaling and plasma membrane trafficking assays. We reveal that the RDY (D130R; R131D) mutation shows a drastic effect on protein trafficking for both human and mouse β2AR. The localization of rI7 RDY mutant in olfactory neurons has never been analyzed *in vivo* nor *in vitro* [21]. In contrast to rI7 RDY activation by octanal, mouse and human RDY mutants maintain functionality *in vitro*. Their EC50s are similar to WT and for both the maximum isoprenaline response is reduced to ~60% (Fig 8). This reduction of the maximum response is most likely a consequence of poor membrane trafficking. A further analysis reveals that the D130R substitution alone blocks membrane trafficking whereas the R131D substitution does not (data not shown). The D130 appears to be a key residue for mouse β2AR trafficking as the mutation D130N also disrupts this process (data not shown).

The neutralizing E268A mutation has the same effect on both human and mouse β2AR. They both have the same EC50 as WT (Fig 8) and their maximum responses are both about 50% of the WT. Conversely, the two proteins show different trafficking phenotypes; mouse E268A retains the capacity to traffic to filopodia (Fig 8) whereas human E268A does not (Fig 8). However, the high sensitivity of the dose response curve assay could not discriminate their differences in plasma membrane trafficking at a single cell level. Finally, the C327R mutation, located at the end of the 7^th^ transmembrane domain, does not affect trafficking to filopodia of both human and mouse β2AR but drastically increases their EC50 for isoprenaline revealing a reduced G-protein coupling efficiency.

In the olfactory system, GPCRs like ORs and β2AR, provide an axonal identity to olfactory neurons that is reflected by the coalescence of like-axons into specific neuropils in the olfactory bulb, called glomeruli [7, 35]. We undertook the analysis of β2AR RDY, C327R and E268A mutants to better understand the *in vivo* significance of these mutants when expressed in olfactory neurons and cause differential axonal identities [8]. The original finding argues that axonal identities vary due to basal and/or ligand-associated activity of the receptor. However, our data open up a third possibility: differential axon identities are a feature of differential plasma membrane trafficking properties. Indeed we have previously shown that a reduction in protein level of an OR is a major determinant for its axonal identity [7, 36]. In addition, it is shown that single amino acid differences in ORs change axonal identities [35]. Thus, it is impossible to uncouple mutations such as RDY, C327R and E268A from directly changing the axonal identity code to their affects on activity and/or receptor trafficking.

### Y326 within the conserved NPxxY motif acts as a retention signal

We reveal that mβ2AR with a Ct-truncation up to and including R328 fails to traffic properly to the plasma membrane, but can be fully rescued by a second mutation Y326A. However, no rescue of isoprenaline response is observed despite robust plasma membrane expression. Unlike RDY, E268A and C327R where all retain some level of isoprenaline-inducible activity, our new loss-of-function mutant (Y326A/ΔCt➔R328) provides an excellent tool to study activity-independent axon guidance in the olfactory system: it traffics properly to the plasma membrane but fully fails to transduce a signal.

We have not characterized what other substitutions of Y326 can rescue the ΔCt➔R328 mutant. However, we have observed that an alanine (A) substitution of all three conserved residues (AAxxA) does not rescue trafficking, thus makes unclear whether there is a structural property and/or a modification of Y326 that occurs in the absence of the Ct.

The NPxxY motif is highly conserved among many GPCRs, and perhaps a mutation in its tyrosine residue might improve plasma membrane trafficking of other non-trafficking GPCRs. However, this improvement in trafficking may also cause a reduction in activity as the Y326A mutation increases isoprenaline-induced EC50 within the intact mβ2AR (Fig 8).

We have tested the effect of altering the NPxxY motif to NPxxA on the non-trafficking M71 OR, with the hypothesis that the Ct of M71 fails to mask the retention thus blocking proper trafficking of the receptor. However, no plasma membrane trafficking was observed in M71 mutants carrying Y289A [11].

### N- and C-termini of a non-trafficking GPCR impair β2AR trafficking

The study of chimeras between mβ2AR and the non-trafficking M71 OR, reveals the negative synergistic effects of the Nt and Ct of M71 on plasma membrane trafficking. The presence of either the Nt or the Ct alone does not impair plasma membrane trafficking of the chimera, indicating that these domains do not modify the secondary structure of the protein. Since it is the Nt and Ct domains on the same protein, which are deleterious for protein trafficking, it is likely that proper protein folding into a seven transmembrane (7TM) structure is dependent on an appropriate interaction between the Nt and Ct of the protein. This is consistent with the proximal relationship between transmembrane domain one (TM1) and TM7 in the 7TM structure and may be a critical feature for how GPCRs normally achieve this confirmation. Conversely, TM1 and TM7 are brought together to form the final tertiary structure by specific chaperones.

Perhaps the Nt and Ct of M71 block the formation of the 7TM structure when M71 is expressed in heterologous cells. Thus, we swapped the Nt and Ct of M71 with those of mβ2AR, generating mβ2AR-Nt/M71/mβ2AR-Ct. This mutant does not traffic to the filopodia of OP6 cells [11]. In addition, the inclusion of the Y289A mutation does not rescue plasma membrane trafficking either [11]. Indeed, our extensive mutant analysis on M71 OR [11] suggested that plasma membrane trafficking of ORs involves a more distributed involvement of the amino acid composition.

In addition, when co-expressing our mβ2AR mutants with the receptor-transporting protein 1S (RTP1S), which is known to improve plasma membrane trafficking of various odorant receptors [37], we do not reveal any significant difference in plasma membrane trafficking for WT mβ2AR::GFP or 17 of our mutants (see pictures and filopodia counts in S4 Fig). Importantly, no rescue is observed for mβ2AR chimeras with M71 Nt and/or Ct. However, several Ct deletion mutants show fewer cells with ≤1 GFP-labeled filopodium. This suggests that the role of RTP1S in improving plasma membrane trafficking is not related to the Nt and Ct interaction that we observe.

### Relationship between trafficking and activity

We observed that mβ2AR::GFP EC50s and maximum responses are relatively refractory to changes in DNA transfected (i.e. a 32 fold reduction is still able to elicit near WT levels; data not shown). These results suggest that changes in EC50 and maximum responses observed in our mβ2AR mutants are solely a function of their trafficking and/or coupling phenotypes. We find that the mβ2AR mutant responses to isoprenaline did not fully correlate with successful trafficking observed for seventeen mutants. Three mutants fail to respond and eleven show a lower maximum response than WT, as low as 20% (black diamonds and green bars in S5 Fig). Hence, even though several mutants traffic effectively to the plasma membrane, they show defects in eliciting responses to isoprenaline.

In contrast, none of the mutants that fail to traffic to the plasma membrane show a maximum response greater than 60% and most of them, six out of ten, fail to respond (red diamonds and red bars in S5 Fig). Overall, only three mutants that successfully traffic to the plasma membrane show similar (2 blue diamonds) or superior (1 violet diamond) maximum response to isoprenaline when compared to WT. Maximum isoprenaline responses for our mutants as high as WT cannot be achieved without successful trafficking. This correlation between filopodia trafficking in OP6 cells and response to isoprenaline in HEK 293 cells indicates that the filopodia assay in OP6 cells is a robust tool to study receptor functionality.

Similar to WT EC50s are observed for mutants that traffic to the plasma membrane (blue diamonds and green bars in S6 Fig) and for three mutants that do not traffic (blue diamonds and red bar in S6 Fig), thus trafficking to the plasma membrane does not always correlate with either maximum response or EC50 to isoprenaline, in the GFP-labeled filopodia assay. This could reflect a distinct behavior of the protein in the two different cell types we use, OP6 cells to address plasma membrane trafficking vs. HEK 293 for FLIPR assay. It would be informative to further characterize some of our mutants with receptor binding assays in order to determine in more detail the association and dissociation rates of the receptor as well as its density at the membrane.

We were surprised that the mutants with EC50s comparable to WT did not always achieve maximum responses (see black box in Fig 8). This suggests a strict default on transduction rather than binding to isoprenaline. An alternate view is that the mutants that poorly traffic to the plasma membrane do not sufficiently deliver enough functional protein to the cell surface to achieve significant responses. In addition, mutants that traffic well to the plasma membrane show log-order differences in EC50 for isoprenaline but achieve the same maximum responses, albeit lower than WT (see differences along vertical axis in Fig 8). This is especially true for our Ct mutants that have a higher affinity for isoprenaline, but have lost their capacity to interact properly with the G-protein signal transduction machinery. Thus, within an *in vivo* setting with sufficient or continuous ligand present, it is unclear if the affinity of the binding is as important to the function of the receptor or if it is the maximum response obtainable (for example, perhaps NtΔ9AA/ΔCt➔Y350/ΔC341 with an EC50 21 times higher than WT and a maximum response of 77% of WT would out perform NtΔ9AA/ mβ2AR/M71-Ct with an EC50 closer to WT (only 6 times higher) but with a lower maximum response of only 34% of WT).

### TM7 and Ct domains of GPCRs are essential for G-protein transduction

All eleven mutants that have higher EC50 (poorer G-protein coupling) compared to WT (EC50/EC50 of WT >2) have mutations in the Ct or the end of the TM7 (mutants in red box in Fig 8). However, the maximum responses for these eleven mutants are variable. Truncations of the Ct to Y350 reduce the maximum response to as low as 67%. The swap of the mβ2AR Ct with the Ct of M71 reduces the maximum response even further to 32%. The ΔCt➔L339 and ΔCt➔E338 truncations reduce the maximum response even more to 27 and 19% of WT, respectively. Point mutations at the end of the 7^th^ TM also increase the EC50 to isoprenaline: the Y326A mutation reduces the maximum response slightly to 89% of mouse WT and human/mouse C327R reduces the maximum response as low as 35% of human WT and to 58% of mouse WT.

Eight other mutations do not affect the EC50 for isoprenaline (EC50/EC50 of WT <2). Those mutations are either located in the Nt of the protein and intracellular domains 2 and 3 (mutants in black box in Fig 8). Neither a reduction of size of the Nt nor the replacement of it with the Nt of M71 affects the maximum response (Fig 8). Thus modifications of the Nt have no discernable effects on signal transduction of the receptor. On the contrary, the double mutant N6A and N15A, which abolishes the only N-glycosylation sites present in mβ2AR, shows an increase in the maximum response (120% of WT, Fig 3). It is unclear if this increase is due to the amino-acid substitutions or due to the lack of N-glycosylation sites. By contrast, the double mutation RDY of the conserved DRY motif reduces the maximum response to isoprenaline to 60% for human RDY and 58% for mouse RDY (Fig 8), without affecting the EC50. The reduction in maximum response is probably due to poor trafficking of the protein (see above). Finally, the mutation E268A in the 3^rd^ intracellular loop of the receptor reduces its maximum response to 51% of both human and mouse WT proteins.

Based on the recent crystallographic studies of human β2AR bound to agonists and fragments of G-proteins, the human β2AR seems to interact with the α subunit of the stimulatory G-protein for adenylyl cyclase (Gαs) via its intracellular loop 2, TM5 and TM6. There are no direct interactions between human β2AR and Gβγ subunits [38]. Therefore, this study did not reveal an interaction of TM7 or the Ct of human β2AR in binding to G-protein. However, as proposed in this study, interactions with Gβy might occur within dimers of human β2AR in the cell and could have been revealed in our study.

## Conclusions

We have characterized over 30 mouse and human β2AR mutants (see S7 Fig). Our extensive set of β2AR mutants reveals major roles for the end of the 7^th^ TM domain and the Ct of the β2AR on activity of the receptor in addition to the already described TM3-TM6 ionic lock model for class A GPCRs [39]. We also reveal for the first time a possible role for Y326 as a trafficking retention signal, as well as a synergistic negative interaction between the Nt and Ct of an OR on trafficking. We describe that mutations can affect both activity and trafficking of the mouse and human β2AR showing the difficulty to dissect those two components when using mutants to study the physiology of the receptor, in particular as a surrogate of an OR. Interestingly, we describe a new mβ2AR loss-of-function mutant for activity that retains proper plasma membrane trafficking but fully fails to respond to isoprenaline. These observations provide a stepping-stone for future mutations using our high-throughput screening assay as well as *in vivo* studies on the physiology of β2AR and ORs.

## Supporting Information

S1 Fig Expression of NtΔ9AA/Y326A/ΔCt➔R328 and Y326A in OP6 cells. (A and B) Both NtΔ9AA/Y326A/ΔCt➔R328 and Y326A express homogenously in OP6 cells and traffic to filopodia (arrow head in magnified pictures).
(EPS)

S2 Fig 2D topology of M71-Nt/mβ2AR/M71-Ct chimeric receptor. The 2D topology of the chimera M71-Nt/mβ2AR/M71-Ct was generated using the online software Topo2 (see Material & Methods). Plasma membrane is symbolized by two horizontal lines with the extracellular compartment on top and intracellular compartment on the bottom. Putative transmembrane domains are located in between those two lines. The Nt and Ct of M71 are depicted as blue circles and the common residues between mβ2AR and M71 present at the limits of those regions are shown in violet circles. The N-linked glycosylation site located in the Nt of the chimera is showed in a black box.
(EPS)

S3 Fig Correlation between The Number Of GFP-Labeled Filopodia Per Cell And The Number Of Cells With ≤1 GFP-Labeled Filopodium. All mutants presented in this study are ranked based on increasing number of GFP-labeled filopodia per cell (1^st^ axis, red and green bars, mean +s.d.). Mutants that fail to traffic to filopodia (p<0.001, one-way ANOVA followed by Scheffe tests, compared to WT) have red bars. Mutants that successfully traffic to filopodia have green bars (p>0.001). On the 2^nd^ axis is showed the number of cells with ≤1 GFP-labeled filopodium, blue diamonds for each mutant. All cells expressing mutants that successfully traffic to filopodia but one (NtΔ9AA/Y326A/ΔCt➔R328 in black box) have more than one GFP-labeled filopodium (blue diamonds at zero). One cell expressing NtΔ9AA/Y326A/ΔCt➔R328 showed no GFP-labeled filopodium. By contrast, for all the mutants classified by our GFP-labeled filopodia assay as non-trafficking mutants (red bars) at least 2/10 cells were observed with ≤1 GFP-labeled filopodium (blue diamonds ≥ 2 and red bars).
(EPS)

S4 Fig Co-expression of mβ2AR mutants with RTP1S has no effect on mutants trafficking. (A) Trafficking to filopodia of mutants that look like an odorant receptor is not affected by co-expression of RTP1S. The average number of GFP-labeled filopodia per cell is showed (bars in graph, mean +s.d.) as well as the number of cells with ≤1 GFP-labeled filopodium (blue diamonds). ***means significantly different from WT, one-way ANOVA followed by Scheffe tests, p<0.001. n.s. means not significantly different from WT, p>0.001. (B, C, D and E) Mutants with shorter Ct that don’t traffic to filopodia are not rescued by co-expression of RTP1S. (F) Trafficking of mutants for Y326 is also unaffected with co-expression of RTP1S. (H and I) In particular no-NxS/Y326A/ΔCt➔R328 and M71-Nt/Y326A/ΔCt➔R328 are not rescued by co-expression of RTP1S. (G) Finally, trafficking of mβ2AR/M71 chimeric receptors is also unchanged by co-expression of RTP1S. (J) In particular the trafficking of the chimeric receptor M71-Nt/mβ2AR/M71-Ct is not rescued by co-expression of RTP1S.
(EPS)

S5 Fig Correlation between trafficking to filopodia and maximum response to isoprenaline. All mutants presented in this study are sorted in increasing number of GFP-labeled filopodia per cell (1^st^ axis, red and green bars, mean +s.d.). Mutants that fail to traffic to filopodia (p<0.001, one-way ANOVA followed by Scheffe tests, compared to WT) have red bars. Mutants that successfully traffic to filopodia have green bars (p>0.001). On the 2^nd^ axis is indicated the maximum response to isoprenaline as percentage of WT. Maximum responses to isoprenaline close to WT (from 97 to 103%) are showed as blue diamonds, higher response (120%) as a magenta diamond, lower responses (from 19 to 90%) as black diamonds and no response as red diamonds. Trafficking does not fully correlate with the maximum of the transduction response obtained.
(EPS)

S6 Fig Correlation between trafficking to filopodia and affinity to isoprenaline. All mutants presented in this study are sorted in increasing number of GFP-labeled filopodia per cell (1^st^ axis, red and green bars, mean +s.d.). Mutants that fail to traffic to filopodia (p<0.001, one-way ANOVA followed by Scheffe tests, compared to WT) have red bars. Mutants that successfully traffic to filopodia have green bars (p>0.001). On the 2^nd^ axis is indicated the EC50 of the mutant for isoprenaline relative to the EC50 of WT on a log scale. Affinity similar to WT (i.e. ratio close to 1, from 0.8 to 2.1) is showed as blue diamonds, and lower affinity compare to WT (i.e. ratio >1, from 4 to 78) is showed as black diamond.
(EPS)

S7 Fig Overview of mβ2AR results. In the summary table is showed a brief description of the GPCR analyzed (1^st^ column) as well as a 2D schematic of the GPCR (2^nd^ column). 3^rd^ column indicates if the receptor successfully traffic to the plasma membrane, as revealed by the GFP-labeled filopodia assay. 4^th^ and 5^th^ columns indicate its transduction efficacy and affinity compare to the WT β2AR::GFP.
(EPS)

S1 Text Nucleotide and amino acid sequences used in mutational analysis. DNA and protein sequences for mouse β2AR, human β2AR, M71, linker, GFP and mCherry and their plasmid names.
(DOCX)
