# Supplementary material for: In Vitro Mutational Analysis of the β2 Adrenergic Receptor, an In Vivo Surrogate Odorant Receptor
Source: PLoS One. 2015 Oct 29;10(10):e0141696. doi: 10.1371/journal.pone.0141696 (PMC4626089; doi:10.1371/journal.pone.0141696)
Supplement: S1 Text — DNA and protein sequences for mouse β2AR, human β2AR, M71, linker, GFP and mCherry and their plasmid names. (DOCX) [file pone.0141696.s008.docx]

# S1 Text. Nucleotide and amino acid sequences used in mutational analysis

## D357 Mouse β_2_AR:

### Amino acid sequence:

MGPHGNDSDFLLAPNGSRAPDHDVTQERDEAWVVGMAILMSVIVLAIVFGNVLVITAIAKFERLQTVTNYFIISLACADLVMGLAVVPFGASHILMKMWNFGNFWCEFWTSIDVLCVTASIETLCVIAVDRYVAITSPFKYQSLLTKNKARVVILMVWIVSGLTSFLPIQMHWYRATHKKAIDCYTEETCCDFFTNQAYAIASSIVSFYVPLVVMVFVYSRVFQVAKRQLQKIDKSEGRFHAQNLSQVEQDGRSGHGLRRSSKFCLKEHKALKTLGIIMGTFTLCWLPFFIVNIVHVIRDNLIPKEVYILLNWLGYVNSAFNPLIYCRSPDFRIAFQELLCLRRSSSKTYGNGYSSNSNGRTDYTGEPNTCQLGQEREQELLCEDPPGMEGFVNCQGTVPSLSVDSQGRNCSTNDSPL

ΔCt🡺R328

NtΔ9AA/ΔCt🡺Y350/ΔC341

Mutated residues analyzed for activity: DRY/RDY, E268A and C327R

C-terminus deletion series I334-L339

### Nucleotide sequence:

atggggccacacgggaacgacagcgacttcttgctggcacccaacggaagccgagcgccagaccacgacgtcactcaggaacgggacgaagcgtgggttgtgggcatggccatcctcatgtcggttatcgtcctggccatcgtgtttggcaacgtgctggtcatcacggccattgccaagttcgagcgactacaaaccgtcaccaactacttcataatctccttggcgtgtgctgatctagtcatgggcctagcggtggtgccgtttggggccagtcacatccttatgaaaatgtggaattttggcaacttctggtgcgagttctggacttccattgatgtgttgtgcgtcacagccagcatcgagaccctgtgcgtgattgcagtggatcgctatgttgctatcacatcgcccttcaagtaccagagcctgctgaccaagaataaggcccgagtggtcatcctgatggtatggattgtatctggccttacctcctttttgcctatccagatgcactggtaccgtgccacccacaagaaagctatcgattgttacaccgaggagacttgctgtgacttcttcacgaaccaggcctacgccatcgcgtcctcgattgtgtctttctacgtgcccctggtggtgatggtctttgtctattcccgggtcttccaggtggccaaaaggcagctgcagaagatagacaaatctgaaggaagattccacgcccaaaacctcagccaggtggagcaggatgggcggagcggccacggactccgaaggtcctccaagttctgcttgaaagagcacaaagccctcaagactttaggcatcatcatgggcacattcaccctctgctggctgcccttcttcattgtcaatatcgtgcacgttatcagggacaacctcatccctaaggaagtttacattctccttaactggttgggctacgtcaactctgccttcaatcctcttatctactgtcggagtccagatttcaggattgcctttcaagagcttctgtgccttcgcaggtcttcttcgaaaacctatgggaacggctactctagcaatagcaacggcagaacggactacacaggggagccaaacacttgtcagctggggcaggagagagaacaggaactgctgtgtgaggatcccccaggcatggaaggctttgtgaactgtcaaggtactgtgcctagccttagcgttgactcccaaggaaggaactgtagtacaaatgactcgccactg

## D356 Human β_2_AR:

### Amino acid sequence:

MGQPGNGSAFLLAPNRSHAPDHDVTQQRDEVWVVGMGIVMSLIVLAIVFGNVLVITAIAKFERLQTVTNYFITSLACADLVMGLAVVPFGAAHILMKMWTFGNFWCEFWTSIDVLCVTASIETLCVIAVDRYFAITSPFKYQSLLTKNKARVIILMVWIVSGLTSFLPIQMHWYRATHQEAINCYANETCCDFFTNQAYAIASSIVSFYVPLVIMVFVYSRVFQEAKRQLQKIDKSEGRFHVQNLSQVEQDGRTGHGLRRSSKFCLKEHKALKTLGIIMGTFTLCWLPFFIVNIVHVIQDNLIRKEVYILLNWIGYVNSGFNPLIYCRSPDFRIAFQELLCLRRSSLKAYGNGYSSNGNTGEQSGYHVEQEKENKLLCEDLPGTEDFVGHQGTVPSDNIDSQGRNCSTNDSLLX

Mutated residues analyzed for activity: DRY/RDY, E268A and C327R

### Nucleotide sequence:

atggggcaacccgggaacggcagcgccttcttgctggcacccaatagaagccatgcgccggaccacgacgtcacgcagcaaagggacgaggtgtgggtggtgggcatgggcatcgtcatgtctctcatcgtcctggccatcgtgtttggcaatgtgctggtcatcacagccattgccaagttcgagcgtctgcagacggtcaccaactacttcatcacttcactggcctgtgctgatctggtcatgggcctggcagtggtgccctttggggccgcccatattcttatgaaaatgtggacttttggcaacttctggtgcgagttttggacttccattgatgtgctgtgcgtcacggccagcattgagaccctgtgcgtgatcgcagtggatcgctactttgccattacttcacctttcaagtaccagagcctgctgaccaagaataaggcccgggtgatcattctgatggtgtggattgtgtcaggccttacctccttcttgcccattcagatgcactggtaccgggccacccaccaggaagccatcaactgctatgccaatgagacctgctgtgacttcttcacgaaccaagcctatgccattgcctcttccatcgtgtccttctacgttcccctggtgatcatggtcttcgtctactccagggtctttcaggaggccaaaaggcagctccagaagattgacaaatctgagggccgcttccatgtccagaaccttagccaggtggagcaggatgggcggacggggcatggactccgcagatcttccaagttctgcttgaaggagcacaaagccctcaagacgttaggcatcatcatgggcactttcaccctctgctggctgcccttcttcatcgttaacattgtgcatgtgatccaggataacctcatccgtaaggaagtttacatcctcctaaattggataggctatgtcaattctggtttcaatccccttatctactgccggagcccagatttcaggattgccttccaggagcttctgtgcctgcgcaggtcttctttgaaggcctatgggaatggctactccagcaacggcaacacaggggagcagagtggatatcacgtggaacaggagaaagaaaataaactgctgtgtgaagacctcccaggcacggaagactttgtgggccatcaaggtactgtgcctagcgataacattgattcacaagggaggaattgtagtacaaatgactcactgctgt

## D358 M71:

### Amino acid sequence:

MTAENQSTVTEFILGGLTNRPELQLPLFLLFLGIYVVTMVGNLGMITLIGLNSQLHTPMYFFLSNLSLVDLCYSSVITPKMLINFVSQRNLISYVGCMSQLYFFLVFVIAECYMLTVMAYDRYVAICQPLLYNIIMSPALCSLLVAFVYAVGLIGSAIETGLMLKLNYCEDLISHYFCDILPLMKLSCSSTYDVEMAVFFLAGFDIIVTSLTVLISYAFILSSILRISSNEGRSKAFSTCSSHFAAVGLFYGSTAFMYLKPSTASSLAQENVASVFYTTVIPMFNPLIYSLRNKEVKTALDKTLRRKVF

N- and C-terminus regions of the odorant receptor M71 used in swap experiments

### Nucleotide sequence:

atgaccgccgagaaccagagcaccgtgaccgagttcatcctgggcggcctgaccaacagacccgagctgcagctgcccctgttcctgctgttcctgggcatctacgtggtgaccatggtgggcaacctgggcatgatcaccctgatcggcctgaacagccagctgcacacccccatgtacttcttcctgagcaacctgagcctggtggacctgtgctacagcagcgtgatcacccccaagatgctgatcaacttcgtgagccagagaaacctgatcagctacgtgggctgcatgagccagctgtacttcttcctggtgttcgtgatcgccgagtgctacatgctgaccgtgatggcctacgacagatacgtggccatctgccagcccctgctgtacaacatcatcatgagccccgccctgtgcagcctgctggtggccttcgtgtacgccgtgggcctgatcggcagcgccatcgagaccggcctgatgctgaagctgaactactgcgaggacctgatcagccactacttctgcgacatcctgcccctgatgaagctgagctgcagcagcacctacgacgtggagatggccgtgttcttcctggccggcttcgacatcatcgtgaccagcctgaccgtgctgatcagctacgccttcatcctgagcagcatcctgagaatcagcagcaacgagggcagaagcaaggccttcagcacctgcagcagccacttcgccgccgtgggcctgttctacggcagcaccgccttcatgtacctgaagcccagcaccgccagcagcctggcccaggagaacgtggccagcgtgttctacaccaccgtgatccccatgttcaaccccctgatctacagcctgagaaacaaggaggtgaagaccgccctggacaagaccctgagaagaaaggtgttctga

## Linker:

### Amino acid sequence:

LINDPPVAT

### Nucleotide sequence:

ttaattaacgatccaccggtcgccacc

## GFP:

### Amino acid sequence:

MVSKGEELFTGVVPILVELDGDVNGHKFSVSGEGEGDATYGKLTLKFICTTGKLPVPWPTLVTTLTYGVQCFSRYPDHMKQHDFFKSAMPEGYVQERTIFFKDDGNYKTRAEVKFEGDTLVNRIELKGIDFKEDGNILGHKLEYNYNSHNVYIMADKQKNGIKVNFKIRHNIEDGSVQLADHYQQNTPIGDGPVLLPDNHYLSTQSALSKDPNEKRDHMVLLEFVTAAGITLGMDELYK

### Nucleotide sequence:

atggtgagcaagggcgaggagctgttcaccggggtggtgcccatcctggtcgagctggacggcgacgtaaacggccacaagttcagcgtgtccggcgagggcgagggcgatgccacctacggcaagctgaccctgaagttcatctgcaccaccggcaagctgcccgtgccctggcccaccctcgtgaccaccctgacctacggcgtgcagtgcttcagccgctaccccgaccacatgaagcagcacgacttcttcaagtccgccatgcccgaaggctacgtccaggagcgcaccatcttcttcaaggacgacggcaactacaagacccgcgccgaggtgaagttcgagggcgacaccctggtgaaccgcatcgagctgaagggcatcgacttcaaggaggacggcaacatcctggggcacaagctggagtacaactacaacagccacaacgtctatatcatggccgacaagcagaagaacggcatcaaggtgaacttcaagatccgccacaacatcgaggacggcagcgtgcagctcgccgaccactaccagcagaacacccccatcggcgacggccccgtgctgctgcccgacaaccactacctgagcacccagtccgccctgagcaaagaccccaacgagaagcgcgatcacatggtcctgctggagttcgtgaccgccgccgggatcactctcggcatggacgagctgtacaagtaa

## mCherry:

### Amino acid sequence:

MVSKGEEDNMAIIKEFMRFKVHMEGSVNGHEFEIEGEGEGRPYEGTQTAKLKVTKGGPLPFAWDILSPQFMYGSKAYVKHPADIPDYLKLSFPEGFKWERVMNFEDGGVVTVTQDSSLQDGEFIYKVKLRGTNFPSDGPVMQKKTMGWEASSERMYPEDGALKGEIKQRLKLKDGGHYDAEVKTTYKAKKPVQLPGAYNVNIKLDITSHNEDYTVEQYERAEGRHSTGGMDELYK

### Nucleotide sequence:

Atggtgagcaagggcgaggaggataacatggccatcatcaaggagttcatgcgcttcaaggtgcacatggagggctccgtgaacggccacgagttcgagatcgagggcgagggcgagggccgcccctacgagggcacccagaccgccaagctgaaggtgaccaagggtggccccctgcccttcgcctgggacatcctgtcccctcagttcatgtacggctccaaggcctacgtgaagcaccccgccgacatccccgactacttgaagctgtccttccccgagggcttcaagtgggagcgcgtgatgaacttcgaggacggcggcgtggtgaccgtgacccaggactcctccctgcaggacggcgagttcatctacaaggtgaagctgcgcggcaccaacttcccctccgacggccccgtaatgcagaagaagaccatgggctgggaggcctcctccgagcggatgtaccccgaggacggcgccctgaagggcgagatcaagcagaggctgaagctgaaggacggcggccactacgacgctgaggtcaagaccacctacaaggccaagaagcccgtgcagctgcccggcgcctacaacgtcaacatcaagttggacatcacctcccacaacgaggactacaccatcgtggaacagtacgaacgcgccgagggccgccactccaccggcggcatggacgagctgtacaagtaa

## D1569-1 RTP1S:

### Amino acid sequence:

MCKSVTTGEWKKVFYEKMEEVKPADSWDFIIDPNLKHNVLAPGWKQYLELHASGRFHCSWCWHTWQSPHVVILFHMYLDKAQRAGSVRMRVFKQLCYECGTARLDESSMLEENIESLVDNLITSLREQCYGERGGHYRIHVASRQDNRRHRGEFCEACQEGIVHWKPSEKLLEEEATTYTFSRAPSPTKPQAETGSGCNFCSIPWCLFWATVLMLIIYLQFSFRTSV

### Nucleotide sequence:

Atgtgtaagagtgtgaccacaggtgagtggaagaaggtcttctacgagaagatggaggaggtgaagccagcggacagctgggacttcatcatagaccccaacctcaagcacaatgtgttggcccctggctggaagcagtacctggaacttcatgcctcaggcaggttccactgttcctggtgctggcacacctggcagtcaccccatgtagtcatcctcttccacatgtacctggacaaggctcagcgcgctggttcggtgcgcatgcgtgtgttcaagcagctctgctacgagtgcggtacagcacggctggatgagtccagcatgctggaggagaacatcgaaagcctggtggacaacctcatcaccagtttgcgagagcagtgctacggggagcgtggtggccactaccgcatccatgtggccagccggcaggacaaccggcgacaccgcggagagttctgcgaggcctgccaggaaggcatcgtgcactggaagcccagtgagaagctgctggaggaggaggcgaccacctacaccttctcccgtgctcccagccccaccaaaccgcaggctgaaacaggctcaggctgcaacttctgctccattccctggtgcttattttgggccacggttttgatgctcatcatctacctgcaattctccttccgtacttctgtctga

**Plasmids names and numbers:**

GFP D1384

M71::GFP D358

gap::GFP D1381

mβ2AR::GFP D357

ΔCt🡺R328 D1188

NtΔ9AA D1780

ΔCt🡺Y350 D1180

NtΔ9AA/ΔCt🡺Y350 D1302

NtΔ9AA/ΔCt🡺Y350/ΔC341 D1384

no-NxS D1556

hβ2AR::GFP D356

human RDY D1749-4

human E268A D1751-1

human C327R D1750-1

mouse RDY D1557

mouse D130R D1586-1

mouse R131D D1587-2

mouse D130N D1585-2

mouse RDY/IREScaGs D1739

mouse E268A D1724

mouse C327R D1722

ΔCt🡺L339 D1589

ΔCt🡺E338 D1591

ΔCt🡺Q337 D1592

ΔCt🡺F336 D1593

ΔCt🡺A335 D1590

ΔCt🡺I334 D1383

Y326A/ΔCt🡺R328 D1599

NtΔ9AA/Y326A/ΔCt🡺R328 D1756

no-NxS/Y326A/ΔCt🡺R328 D1748

M71-Nt/Y326A/ΔCt🡺R328 D1721

Y326A D1755

M71-Nt/mβ2AR D1711

mβ2AR/M71-Ct D1194

M71-Nt/mβ2AR/M71-Ct D1712

NtΔ9AA/mβ2AR/M71-Ct D1313

M71-Nt/mβ2AR::mcherry D1757

mβ2AR/M71-Ct::mcherry D1758

RTP1S D1569
